# Age-specific associations between underlying health conditions and hospitalisation, death and in-hospital death among confirmed COVID-19 cases: a multi-country study based on surveillance data, June to December 2020

**DOI:** 10.2807/1560-7917.ES.2022.27.35.2100883

**Published:** 2022-09-01

**Authors:** Tjede Funk, Francesco Innocenti, Joana Gomes Dias, Lina Nerlander, Tanya Melillo, Charmaine Gauci, Jackie M Melillo, Patrik Lenz, Helena Sebestova, Pavel Slezak, Iva Vlckova, Jacob Dag Berild, Camilla Mauroy, Elina Seppälä, Ragnhild Tønnessen, Anne Vergison, Joël Mossong, Silvana Masi, Laetitia Huiart, Gillian Cullen, Niamh Murphy, Lois O’Connor, Joan O’Donnell, Piers Mook, Richard G Pebody, Nick Bundle

**Affiliations:** 1 European Centre for Disease Prevention and Control (ECDC), Stockholm, Sweden; 2 Epidemiology Unit, Regional Health Agency of Tuscany, Florence, Italy; 3 Infectious Disease Prevention and Control Unit, Superintendence of Public Health, Gwardamanġa, Malta; 4 Superintendence of Public Health, Valletta, Malta; 5 Department of Biostatistics, National Institute of Public Health, Prague, Czechia; 6 Department of Infectious Diseases Epidemiology, National Institute of Public Health, Prague, Czechia; 7 Division of Infection Control, Norwegian Institute of Public Health, Oslo, Norway; 8 European Programme for Intervention Epidemiology Training (EPIET), European Centre for Disease Prevention and Control (ECDC), Stockholm, Sweden; 9 European Public Health Microbiology Training Programme (EUPHEM), European Centre for Disease Prevention and Control (ECDC), Stockholm, Sweden; 10 Health Directorate, Luxembourg, Luxembourg; 11 HSE - Health Protection Surveillance Centre, Dublin, Ireland; 12 World Health Organization Regional Office for Europe, Copenhagen, Denmark

**Keywords:** SARS-CoV-2, COVID-19, underlying health conditions, hospitalisation, death, in-hospital death

## Abstract

**Background:**

Underlying conditions are risk factors for severe COVID-19 outcomes but evidence is limited about how risks differ with age.

**Aim:**

We sought to estimate age-specific associations between underlying conditions and hospitalisation, death and in-hospital death among COVID-19 cases.

**Methods:**

We analysed case-based COVID-19 data submitted to The European Surveillance System between 2 June and 13 December 2020 by nine European countries. Eleven underlying conditions among cases with only one condition and the number of underlying conditions among multimorbid cases were used as exposures. Adjusted odds ratios (aOR) were estimated using 39 different age-adjusted and age-interaction multivariable logistic regression models, with marginal means from the latter used to estimate probabilities of severe outcome for each condition–age group combination.

**Results:**

Cancer, cardiac disorder, diabetes, immunodeficiency, kidney, liver and lung disease, neurological disorders and obesity were associated with elevated risk (aOR: 1.5–5.6) of hospitalisation and death, after controlling for age, sex, reporting period and country. As age increased, age-specific aOR were lower and predicted probabilities higher. However, for some conditions, predicted probabilities were at least as high in younger individuals with the condition as in older cases without it. In multimorbid patients, the aOR for severe disease increased with number of conditions for all outcomes and in all age groups.

**Conclusion:**

While supporting age-based vaccine roll-out, our findings could inform a more nuanced, age- and condition-specific approach to vaccine prioritisation. This is relevant as countries consider vaccination of younger people, boosters and dosing intervals in response to vaccine escape variants.

## Introduction

Already early on in the coronavirus disease (COVID-19) pandemic, different factors were identified as being associated with an increased risk of severe COVID-19, including older age, male sex and underlying conditions such as diabetes, chronic liver disease or chronic heart disease [1-4]. Chronic disease burden is known to increase with age [5-7]. Accordingly, many countries have taken an age-based approach to roll out vaccines against COVID-19, with additional priority being given to people of younger ages according to their occupation, risk of exposure or the presence of certain underlying conditions [8,9].

In European Union/European Economic Area (EU/EEA) countries, COVID-19 vaccine boosters have been offered to the adult population and vaccination has been approved by the European Medicines Agency for children as young as 5 years in 2021 [10]. Informed national policy decisions about which populations to target with the primary course or additional vaccine doses, and at what interval, rely on risk–benefit assessments based on age-specific estimates of COVID-19 severity. Furthermore, it is recommended that prioritisation according to underlying conditions be based on identification of the additional risk conferred by individual conditions [9]. There is limited evidence on the strength of association between underlying conditions and severe COVID-19 in different age groups, and on the contribution of individual conditions to the risk of severe disease.

This study sought to estimate relative and absolute effects of individual underlying conditions on hospitalisation, death and in-hospital death in different age groups, among COVID-19 cases reported by a subset of EU/EEA countries between June and December 2020. This period represented a time when testing was fairly well established in most EU/EEA countries and largely predated both the roll-out of vaccines and the widespread transmission of severe acute respiratory syndrome coronavirus 2 (SARS-CoV-2) variants of concern in the EU/EEA.

## Methods

### Data sources and periods

Since spring 2020, all EU/EEA countries have been submitting case-based and/or aggregated data on COVID-19 on a weekly basis to The European Surveillance System (TESSy). We extracted case-based COVID-19 data submitted to TESSy by nine countries (Czechia, Finland, Ireland, Italy, Luxembourg, Malta, Norway, Poland and Slovakia) [11]. We included data from countries that had consistently reported data on the variables used in this study until the reporting week ending 10 January 2021. Cases were excluded if reported in the 4 weeks before this date (to minimise possible misclassification of fatal outcome) or during the early phase of the pandemic (when limited testing capacity may have led to an over-representation of severe cases). Consequently, our principal analysis used cases reported in the period from 2 June to 13 December 2020.

### Outcome variables

The three binary outcomes were hospitalisation among all cases (hereafter hospitalisation), death among all cases, irrespective of their hospitalisation status (hereafter death) and death among the subset of cases who had been hospitalised (hereafter in-hospital deaths). We assumed that the threshold for admission to hospital was less variable between countries or over time than the chance of becoming a case, which may have been prone to ascertainment bias due to differential or changing testing strategies. Despite the smaller sample size when restricting to hospitalised cases, analyses using in-hospital death as the outcome were used to compare and validate findings for the ‘death’ variable.

### Exposure variables

Eleven underlying conditions included in the coded value list in case-based reporting in TESSy were included as main exposures in this analysis: asthma, cancer (coded value in TESSy: cancer, malignancy), cardiac disorder (cardiac disorder, excluding hypertension), diabetes, immune deficiency disorder (HIV/other immune deficiency disorders), hypertension, kidney disease (kidney-related condition, renal disease), liver disease (liver-related condition, liver disease), lung disease (chronic lung disease, excluding asthma), neurological disorders (neuromuscular disorder, chronic neurological conditions) and obesity [11]. We created two additional categorical exposure variables based on the number of conditions a case had recorded: *number of underlying conditions (1,2,* ≥ *3)* and *the presence of* *any underlying condition (* ≥ *1),* resulting in a total of 13 exposure variables. For each exposure, the same comparator group was used, comprising all cases reported as having no underlying conditions. For the 11 main underlying conditions, the exposure group comprised cases with *only* that condition, to reduce potential confounding, interactions and collinearity between conditions in multimorbid patients. The number of people with each condition varied. Since each condition was analysed separately, a different number of cases was included in the analyses for a given exposure condition. Covariables were age (0–19 years and then 10-year groups to ≥ 80 years), sex (male/female), reporting period (two periods to reflect different stages of the pandemic in the EU/EEA: June to September and October to December 2020) and reporting country.

### Missing or incomplete data

Fatal cases whose cause of death was unknown or other than COVID-19 were excluded. Cases coded as still being on treatment at the time of the analysis were coded as alive. Completeness of reporting of deaths to TESSy was assessed through comparison with publicly reported official data [12]. In three countries (Finland, Ireland and Slovakia), cases for whom fatal outcome was unknown were recoded as alive as ≥ 90% of the official death totals had been reported to TESSy and ≥ 10% of cases had an unknown outcome. We considered this a conservative approach since increasing the size of the denominator (alive) in analyses with death as the outcome would probably lead to an underestimation of the effect. This approach was not possible for hospitalisation status because an external comparator data source was not available. We generated a complete case series for analysis by excluding all cases with unknown or missing information on any of the outcomes, exposures or covariables. The distributions of outcome, exposure and co-variables across all reported cases, those excluded because of missing information and those included in the analysis, were inspected to assess possible ascertainment bias.

### Statistical analysis

We followed the same procedure for each of 39 combinations, reflecting the 13 exposure conditions with three outcomes. We calculated crude risks for each outcome (number of cases with outcome divided by the number of cases) for each value of the main exposure and covariables. Differences in the distribution of cases across these variables were assessed using a chi-squared test and crude measures of association (odds ratios (OR)) between each variable and the outcome using univariable logistic regression.

Adjusted OR (aOR) for the association between each exposure condition and outcome were estimated using two multivariable logistic regression models. The first, an age-adjusted model, controlled for all covariables, including age group, with p values estimated for independent variables using a likelihood ratio test (LRT). The second, an age-interaction model, included an interaction between the main exposure condition and age with all other covariables. We assessed the global effect of the interaction by comparing these models using a LRT. Irrespective of the interaction LRT p value, we used the age-interaction models to estimate age-specific aOR for each combination of exposure and outcome.

In addition, using the function 'ggemmeans' from the R package ggeffects, marginal means were estimated from the age-interaction models to estimate the predicted probability of the outcome for each level of condition and age group, marginalised over the levels of each covariable [13]. To decide how best to control for differences between reporting countries, we compared estimates and standard errors from models using the country as a fixed effect with those from models with cluster standard errors on reporting country. We chose to use the former (i.e. fixed effects) as there was no important difference between the two approaches. All analyses were conducted in R version v4.0.4 (R Foundation, Vienna, Austria).

### Sensitivity analysis

A sensitivity analysis was conducted repeating the above steps for cases reported between 6 February and 13 December 2020 to assess the impact of the decision to exclude cases from the earlier part of the pandemic in the principal analysis. Findings were visually compared with those from the principal analysis.

## Results

Between 2 June and 13 December 2020, the included countries reported 2,614,881 COVID-19 cases, 763,674 (29%) of whom were included in the complete case series for analysis and 638,213 of these included cases belonged to the reference population (Table 1). Most exclusions were cases without data on underlying conditions (68%). The proportion of hospitalised or fatal cases was slightly higher among the cases used in the analysis and there was no difference in the age and sex distribution. In the Supplementary Tables we provide a detailed breakdown of the characteristics of cases included in the primary analysis as well as the distribution of outcome and exposure variables across cases included and cases dropped from the analysis.

**Table 1:** Distribution of study population by age and sex with crude risk for each underlying condition and outcome, COVID-19 cases, EU/EEA^a^, 2 June–13 December 2020

Underlying condition	Population size^b^	Sex (%)	Age groups in years (%)								Outcome					
											Hospitalisation		Death		In-hospital death	
	Total	Male	< 20	20–29	30–39	40–49	50–59	60–69	70–79	≥ 80	n	Crude risk (%)	n	Crude risk (%)	n	Crude risk (%)
No underlying condition (reference)	638,213	47.8	13.7	14.4	15.3	18.3	17.3	9.5	5.7	5.7	39,350	6.2	8,942	1.4	6,310	16.0
Asthma	1,525	43.9	15.1	19.2	19.0	20.3	14.8	7.7	2.7	1.2	100	6.6	5	0.3	4	4.0
Cancer	17,834	46.1	0.9	1.3	3.1	8.5	19.0	20.9	23.6	22.5	4,713	26.4	1,753	9.8	1,377	29.2
Cardiac disorder	39,659	51.4	0.8	0.8	1.9	7.0	18.7	21.5	20.2	29.1	11,899	30.0	4,256	10.7	3,613	30.4
Diabetes	23,995	52.4	1.1	1.6	4.6	8.0	14.7	20.8	23.3	25.9	7,613	31.7	2,663	11.1	2,213	29.1
Hypertension	2,746	48.4	1.1	1.3	5.3	17.7	31.5	24.4	12.1	6.6	241	8.8	16	0.6	13	5.4
Immune deficiency disorder	749	40.9	6.0	10.9	14.4	19.1	25.8	12.6	7.9	3.3	133	17.8	32	4.3	29	21.8
Kidney disease	1,922	57.0	3.6	3.6	7.1	11.2	16.8	19.5	19.2	19.0	759	39.5	256	13.3	230	30.3
Liver disease	1,283	55.8	1.5	3.2	9.7	21.0	30.3	20.4	8.4	5.4	201	15.7	48	3.7	31	15.4
Lung disease	11,358	48.0	12.8	12.1	10.7	15.8	18.0	12.2	9.1	9.2	1,696	14.9	483	4.3	392	23.1
Neurological disorders	3,370	41.6	5.2	5.6	8.5	10.9	13.7	11.5	15.4	29.1	847	25.1	440	13.1	332	39.2
Obesity	1,500	50.4	4.7	13.9	18.5	23.4	23.7	9.7	4.0	2.1	126	8.4	14	0.9	12	9.5
Any underlying condition (≥ 1)	125,461	49.4	2.9	4.5	6.0	10.1	17.9	18.9	18.3	21.5	32,305	25.7	10,878	8.7	9,000	27.9
Number of underlying conditions (1)	113,190	49.5	2.9	4.4	5.9	10.0	17.8	18.8	18.3	21.9	29,006	25.6	10,034	8.9	8,310	28.6
Number of underlying conditions (2)	8,841	47.6	3.4	6.1	8.0	12.7	19.3	19.7	16.4	14.5	1,952	22.1	435	4.9	366	18.8
Number of underlying conditions (≥ 3)	3,430	48.9	0.8	1.3	2.7	7.3	16.2	21.1	24.4	26.1	1,347	39.3	409	11.9	324	24.1

COVID-19: coronavirus disease; EU/EEA: European Union/European Economic Area.

^a^ Countries included in this analysis: Czechia, Finland, Ireland, Italy, Luxembourg, Malta, Norway, Poland and Slovakia.

^b^ Number of individuals included in the analysis.

### Overall findings

After controlling for age, sex, reporting period and reporting country in the age-adjusted models, cases with an underlying condition had between 1.50 and 5.63 times higher odds of severe outcomes than cases without underlying condition (Table 2, Table 3, Table 4). The only exceptions to this were asthma and hypertension. Only five deaths were reported among cases with asthma and there was no association with either fatal outcome (Tables 3 and 4). Hypertension was not associated with hospitalisation in the age-adjusted model but was a risk factor in multiple age strata (Table 2). Cases with hypertension had reduced odds of fatal outcomes in the age-adjusted model but no age-specific associations were present (Tables 3 and 4).

**Table 2:** Age-adjusted and age stratum-specific associations between underlying condition and hospitalisation, COVID-19 cases, EU/EEA^a^, 2 June–13 December 2020

Underlying condition	Age-adjusted model		Age-interaction model, stratum-specific aOR (95% CI)								
	aOR (95% CI)	p value^b^	< 20 years	20–29 years	30–39 years	40–49 years	50–59 years	60–69 years	70–79 years	≥ 80 years	Interaction p value^b^
Asthma	1.83 (1.44–2.33)	< 0.0001	1.99 (0.87–4.56)	1.17 (0.48–2.87)	1.96 (1.08–3.55)	2.23 (1.36–3.65)	2.36 (1.51–3.70)	1.25 (0.72–2.19)	1.68 (0.83–3.38)	1.95 (0.75–5.07)	0.64
Cancer	2.01 (1.93–2.09)	< 0.0001	36.81 (26.02–52.08)	6.20 (3.93–9.77)	4.81 (3.62–6.39)	3.75 (3.15–4.47)	2.98 (2.69–3.31)	2.47 (2.28–2.68)	1.78 (1.66–1.90)	1.42 (1.33–1.53)	< 0.0001
Cardiac disorder	2.13 (2.07–2.18)	< 0.0001	5.47 (3.53–8.50)	4.37 (2.82–6.79)	4.74 (3.75–6.00)	3.76 (3.33–4.25)	3.12 (2.92–3.34)	2.12 (2.00–2.24)	1.86 (1.76–1.96)	1.85 (1.77–1.94)	< 0.0001
Diabetes	2.53 (2.45–2.61)	< 0.0001	8.75 (5.74–13.34)	6.46 (4.53–9.21)	6.17 (5.09–7.47)	5.59 (4.90–6.38)	4.82 (4.43–5.25)	2.97 (2.78–3.18)	2.20 (2.07–2.33)	1.68 (1.59–1.78)	< 0.0001
Immune deficiency disorder	3.58 (2.91–4.41)	< 0.0001	5.85 (1.80–19.05)	2.50 (0.78–7.98)	6.13 (3.40–11.05)	5.06 (3.07–8.36)	4.45 (3.08–6.43)	2.71 (1.70–4.32)	3.05 (1.81–5.16)	0.92 (0.38–2.24)	0.009
Hypertension	1.03 (0.88–1.20)	0.72	18.65 (6.44–54.07)	2.26 (0.30–16.73)	2.48 (1.15–5.35)	1.52 (0.94–2.45)	1.40 (1.05–1.87)	0.72 (0.54–0.97)	0.75 (0.55–1.03)	1.22 (0.87–1.71)	< 0.0001
Kidney disease	4.86 (4.37–5.40)	< 0.0001	9.95 (4.51–21.92)	9.57 (4.82–18.99)	9.75 (6.40–14.86)	8.76 (6.30–12.18)	7.61 (5.97–9.69)	4.98 (4.02–6.15)	3.54 (2.86–4.37)	2.97 (2.40–3.67)	< 0.0001
Liver disease	2.14 (1.82–2.52)	< 0.0001	10.64 (2.43–46.57)	4.69 (1.43–15.40)	3.18 (1.60–6.31)	4.53 (3.14–6.52)	2.21 (1.61–3.03)	2.43 (1.82–3.24)	1.28 (0.83–1.96)	0.66 (0.37–1.17)	< 0.0001
Lung disease	2.18 (2.05–2.31)	< 0.0001	1.60 (1.09–2.34)	1.94 (1.41–2.69)	2.63 (2.05–3.37)	2.70 (2.25–3.24)	2.58 (2.25–2.95)	2.40 (2.11–2.73)	2.00 (1.76–2.28)	1.74 (1.53–1.97)	< 0.0001
Neurological disorders	2.20 (2.02–2.41)	< 0.0001	8.34 (5.02–13.87)	5.82 (3.41–9.94)	5.21 (3.54–7.65)	4.92 (3.59–6.74)	4.01 (3.15–5.12)	3.23 (2.59–4.03)	1.72 (1.42–2.07)	1.46 (1.27–1.66)	< 0.0001
Obesity	1.83 (1.44–2.34)	< 0.0001	1.19 (0.16–8.65)	3.14 (1.66–5.92)	1.12 (0.52–2.41)	2.05 (1.29–3.25)	2.07 (1.42–3.03)	1.29 (0.77–2.16)	1.59 (0.86–2.95)	2.54 (1.21–5.34)	0.29
Any underlying condition (≥ 1)	2.35 (2.30–2.39)	< 0.0001	5.16 (4.42–6.02)	3.14 (2.73–3.62)	3.79 (3.44–4.18)	3.89 (3.64–4.16)	3.32 (3.17–3.48)	2.45 (2.35–2.55)	2.02 (1.94–2.09)	1.77 (1.71–1.83)	< 0.0001
Number of underlying conditions (1)^c^	2.29 (2.25–2.34)	< 0.0001	5.11 (4.35–6.00)	3.11 (2.68–3.62)	3.79 (3.42–4.19)	3.81 (3.55–4.09)	3.31 (3.16–3.47)	2.41 (2.31–2.51)	1.95 (1.88–2.03)	1.72 (1.66–1.78)	< 0.0001
Number of underlying conditions (2)^c^	2.61 (2.45–2.77)	< 0.0001	6.04 (3.49–10.43)	4.22 (2.78–6.41)	4.24 (3.13–5.75)	4.51 (3.68–5.53)	3.03 (2.61–3.52)	2.36 (2.10–2.66)	2.17 (1.93–2.43)	2.42 (2.15–2.73)	< 0.0001
Number of underlying conditions (≥ 3)^c^	4.58 (4.22–4.97)	< 0.0001	28.16 (10.42–76.05)	10.58 (4.10–27.31)	8.79 (4.83–15.99)	9.27 (6.64–12.95)	6.25 (5.07–7.70)	4.86 (4.14–5.71)	4.70 (4.06–5.44)	3.11 (2.70–3.58)	< 0.0001

aOR: adjusted odds ratio; CI: confidence interval; COVID-19: coronavirus disease; EU/EEA: European Union/European Economic Area; NA: not available as no outcome occurred within the age group.

^a^ Countries included in this analysis: Czechia, Finland, Ireland, Italy, Luxembourg, Malta, Norway, Poland and Slovakia.

^b^ p values based on likelihood ratio test.

^c^ Values are based on the same model. The reference group for all models was COVID-19 cases with no underlying conditions.

**Table 3:** Age-adjusted and age stratum-specific associations between underlying condition and death, COVID-19 cases, EU/EEA^a^, 2 June–13 December 2020

Underlying condition	Age-adjusted model		Age-interaction model, stratum-specific aOR (95% CI)								
	aOR (95% CI)	p value^b^	< 20 years	20–29 years	30–39 years	40–49 years	50–59 years	60–69 years	70–79 years	≥ 80 years	Interaction p value^b^
Asthma	0.93 (0.36–2.38)	0.88	NA	NA	NA	NA	NA	1.67 (0.40–7.00)	1.57 (0.36–6.83)	0.44 (0.06–3.52)	0.88
Cancer	1.92 (1.81–2.04)	< 0.0001	438.59 (145.45–1,322.54)	69.29 (8.06–595.71)	33.55 (14.83–75.92)	18.87 (12.04–29.59)	8.83 (6.83–11.41)	5.11 (4.43–5.91)	2.15 (1.93–2.38)	1.24 (1.15–1.35)	< 0.0001
Cardiac disorder	1.94 (1.86–2.03)	< 0.0001	NA	52.19 (6.08–448.29)	24.64 (10.90–55.69)	7.69 (4.75–12.46)	4.51 (3.57–5.69)	3.11 (2.75–3.52)	2.09 (1.92–2.27)	1.68 (1.60–1.77)	< 0.0001
Diabetes	2.02 (1.93–2.13)	< 0.0001	NA	NA	7.83 (2.40–25.51)	9.48 (5.65–15.90)	8.30 (6.52–10.56)	4.00 (3.49–4.58)	2.46 (2.25–2.69)	1.48 (1.39–1.58)	< 0.0001
Immune deficiency disorder	4.60 (3.06–6.90)	< 0.0001	NA	NA	27.11 (3.67–200.55)	17.97 (4.38–73.78)	8.37 (3.08–22.75)	6.72 (3.23–14.00)	4.40 (2.31–8.38)	1.37 (0.50–3.72)	0.067
Hypertension	0.45 (0.26–0.77)	0.0014	NA	NA	NA	NA	NA	0.17 (0.02–1.25)	0.56 (0.24–1.30)	0.51 (0.25–1.05)	0.89
Kidney disease	4.11 (3.52–4.80)	< 0.0001	NA	NA	57.04 (17.28–188.30)	15.18 (4.77–48.31)	17.72 (10.40–30.21)	10.30 (7.48–14.19)	4.06 (3.11–5.31)	2.48 (1.98–3.10)	< 0.0001
Liver disease	2.30 (1.67–3.16)	< 0.0001	NA	NA	42.82 (10.16–180.45)	7.80 (1.91–31.80)	7.63 (3.89–14.94)	4.27 (2.55–7.12)	1.09 (0.51–2.37)	1.02 (0.54–1.91)	< 0.0001
Lung disease	2.12 (1.91–2.36)	< 0.0001	NA	14.52 (1.69–124.40)	14.04 (5.89–33.46)	3.39 (1.38–8.34)	3.57 (2.23–5.69)	3.44 (2.64–4.48)	2.32 (1.91–2.82)	1.67 (1.45–1.93)	< 0.0001
Neurological disorders	3.15 (2.80–3.54)	< 0.0001	127.56 (26.28–619.10)	195.83 (37.72–1016.81)	19.42 (4.65–81.19)	25.50 (12.30–52.87)	17.02 (10.70–27.09)	8.02 (5.73–11.23)	3.63 (2.87–4.60)	2.30 (1.99–2.66)	< 0.0001
Obesity	5.63 (2.27–13.97)	0.00017	NA	NA	NA	NA	10.62 (2.88–39.15)	8.54 (2.50–29.13)	4.24 (1.04–17.20)	3.99 (1.06–15.05)	0.78
Any underlying condition (≥ 1)	2.14 (2.07–2.21)	< 0.0001	33.77 (12.57–90.75)	24.04 (7.33–78.82)	16.63 (10.50–26.36)	9.35 (7.04–12.44)	6.55 (5.60–7.65)	4.08 (3.72–4.46)	2.33 (2.19–2.48)	1.61 (1.55–1.68)	< 0.0001
Number of underlying conditions (1)^c^	2.07 (2.01–2.14)	< 0.0001	33.82 (12.25–93.34)	23.53 (6.81–81.34)	16.39 (10.18–26.41)	9.39 (7.01–12.57)	6.36 (5.43–7.46)	3.91 (3.56–4.28)	2.26 (2.13–2.41)	1.56 (1.50–1.63)	< 0.0001
Number of underlying conditions (2)^c^	3.02 (2.67–3.41)	< 0.0001	63.32 (7.73–518.54)	60.70 (7.06–522.30)	34.68 (13.47–89.32)	9.60 (3.88–23.72)	9.69 (6.28–14.97)	5.65 (4.38–7.28)	2.56 (2.06–3.18)	2.42 (2.06–2.84)	< 0.0001
Number of underlying conditions (≥ 3)^c^	5.52 (4.82–6.32)	< 0.0001	NA	NA	NA	31.98 (10.02–102.11)	18.67 (10.35–33.70)	12.58 (9.41–16.81)	6.50 (5.25–8.03)	3.79 (3.18–4.53)	< 0.0001

aOR: adjusted odds ratio; CI: confidence interval; COVID-19: coronavirus disease; EU/EEA: European Union/European Economic Area; NA: not available as no outcome occurred within the age group.

^a^ Countries included in this analysis: Czechia, Finland, Ireland, Italy, Luxembourg, Malta, Norway, Poland and Slovakia.

^b^ p values based on likelihood ratio test.

^c^ Values are based on the same model. The reference group for all models was COVID-19 cases with no underlying conditions.

**Table 4:** Age-adjusted and age stratum-specific associations between underlying condition and in-hospital death, COVID-19 cases, EU/EEA^a^, 2 June–13 December 2020

Underlying condition	Age-adjusted model		Age-interaction model, stratum-specific aOR (95% CI)								
	aOR (95% CI)	p value^b^	< 20 years	20–29 years	30–39 years	40–49 years	50–59 years	60–69 years	70–79 years	≥ 80 years	Interaction p value^b^
Asthma	0.68 (0.23–1.98)	0.46	NA	NA	NA	NA	NA	2.32 (0.49–11.02)	0.54 (0.07–4.55)	0.49 (0.06–4.16)	0.82
Cancer	1.54 (1.43–1.66)	< 0.0001	20.47 (5.68–73.76)	14.91 (1.58–140.33)	11.32 (4.37–29.31)	7.81 (4.71–12.95)	4.28 (3.19–5.74)	2.64 (2.21–3.14)	1.53 (1.35–1.74)	1.06 (0.95–1.18)	< 0.0001
Cardiac disorder	1.52 (1.44–1.60)	< 0.0001	NA	16.25 (1.73–152.87)	6.95 (2.73–17.72)	2.51 (1.49–4.22)	1.80 (1.40–2.32)	1.83 (1.59–2.11)	1.52 (1.38–1.68)	1.42 (1.33–1.52)	0.00047
Diabetes	1.50 (1.41–1.60)	< 0.0001	NA	NA	2.02 (0.60–6.86)	2.24 (1.28–3.90)	2.47 (1.90–3.23)	1.86 (1.59–2.17)	1.61 (1.45–1.80)	1.26 (1.16–1.38)	< 0.0001
Immune deficiency disorder	3.09 (1.95–4.90)	< 0.0001	NA	NA	9.13 (1.11–75.00)	2.76 (0.36–21.22)	2.88 (1.00–8.27)	4.15 (1.75–9.83)	3.04 (1.42–6.48)	1.26 (0.28–5.65)	0.9
Hypertension	0.49 (0.26–0.93)	0.021	NA	NA	NA	NA	NA	0.21 (0.03–1.54)	0.76 (0.27–2.09)	0.54 (0.22–1.33)	0.87
Kidney disease	2.41 (2.02–2.87)	< 0.0001	NA	NA	10.67 (2.97–38.31)	2.91 (0.88–9.64)	3.88 (2.12–7.10)	3.79 (2.62–5.49)	2.13 (1.56–2.90)	1.83 (1.38–2.42)	0.024
Liver disease	1.71 (1.13–2.60)	0.016	NA	NA	32.35 (6.02–173.86)	2.93 (0.68–12.57)	3.72 (1.54–8.94)	2.38 (1.28–4.44)	0.45 (0.14–1.51)	0.77 (0.26–2.27)	0.0056
Lung disease	1.60 (1.41–1.82)	< 0.0001	NA	NA	9.14 (3.56–23.49)	1.36 (0.49–3.79)	1.68 (1.01–2.81)	2.05 (1.52–2.77)	1.51 (1.19–1.92)	1.45 (1.19–1.76)	0.048
Neurological disorders	3.08 (2.62–3.62)	< 0.0001	26.50 (4.62–151.86)	23.57 (2.45–226.71)	2.91 (0.38–22.43)	10.08 (4.49–22.65)	6.61 (3.83–11.43)	4.36 (2.90–6.55)	3.03 (2.21–4.15)	2.34 (1.88–2.90)	0.00018
Obesity	3.21 (1.09–9.42)	0.031	NA	NA	NA	NA	4.21 (0.83–21.46)	7.89 (1.80–34.58)	3.35 (0.67–16.70)	1.62 (0.32–8.17)	0.7
Any underlying condition (≥ 1)	1.63 (1.56–1.69)	< 0.0001	8.22 (2.66–25.46)	6.59 (1.63–26.56)	6.48 (3.75–11.19)	3.25 (2.36–4.46)	2.56 (2.15–3.05)	2.21 (2.00–2.46)	1.62 (1.50–1.74)	1.37 (1.30–1.45)	< 0.0001
Number of underlying conditions (1)^c^	1.58 (1.51–1.64)	< 0.0001	8.05 (2.52–25.71)	5.62 (1.25–25.33)	6.15 (3.51–10.78)	3.19 (2.30–4.41)	2.46 (2.06–2.95)	2.08 (1.87–2.32)	1.58 (1.46–1.70)	1.34 (1.27–1.42)	< 0.0001
Number of underlying conditions (2)^c^	2.30 (2.00–2.66)	< 0.0001	15.67 (1.69–145.65)	24.91 (2.66–233.64)	16.63 (5.42–51.06)	3.94 (1.54–10.04)	4.54 (2.73–7.58)	3.98 (2.98–5.30)	1.86 (1.44–2.39)	1.87 (1.53–2.27)	< 0.0001
Number of underlying conditions (≥ 3)^c^	3.29 (2.79–3.87)	< 0.0001	NA	NA	NA	7.84 (2.34–26.24)	5.57 (2.92–10.62)	5.86 (4.23–8.13)	3.36 (2.63–4.30)	2.41 (1.93–3.02)	< 0.0001

aOR: adjusted odds ratio; CI: confidence interval; COVID-19: coronavirus disease; EU/EEA: European Union/European Economic Area; NA: not available as no outcome occurred within the age group;

^a^ Countries included in this analysis: Czechia, Finland, Ireland, Italy, Luxembourg, Malta, Norway, Poland and Slovakia.

^b^ p values based on likelihood ratio test;

^c^ Values are based on the same model. The reference group for all models were COVID-19 cases with no underlying condition.

**Figure 1:**
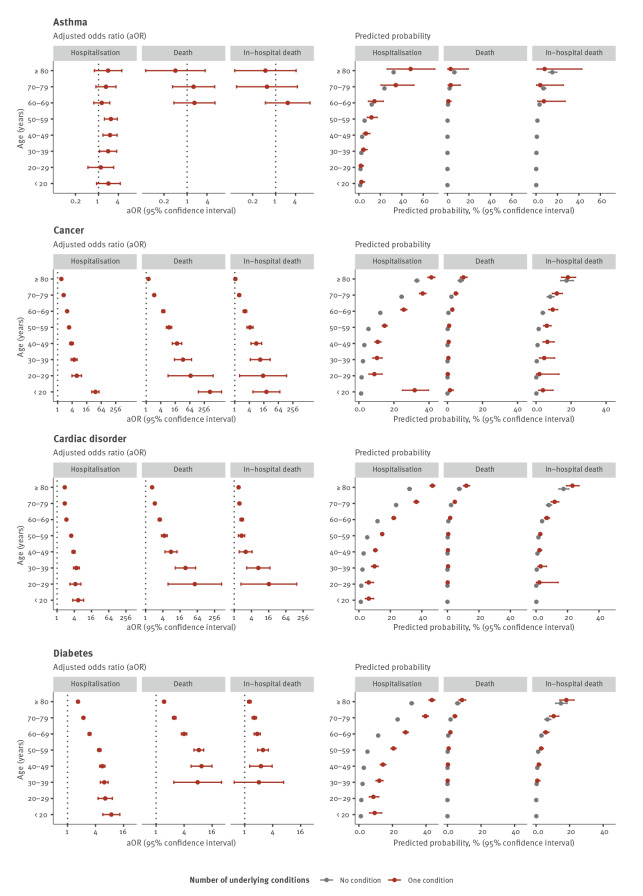
Adjusted odds ratios and predicted probabilities of hospitalisation, death and in-hospital death for the first set of underlying condition compared with COVID-19 cases without an underlying condition, EU/EEA^a^, 2 June–13 December 2020^b^

**Figure 2:**
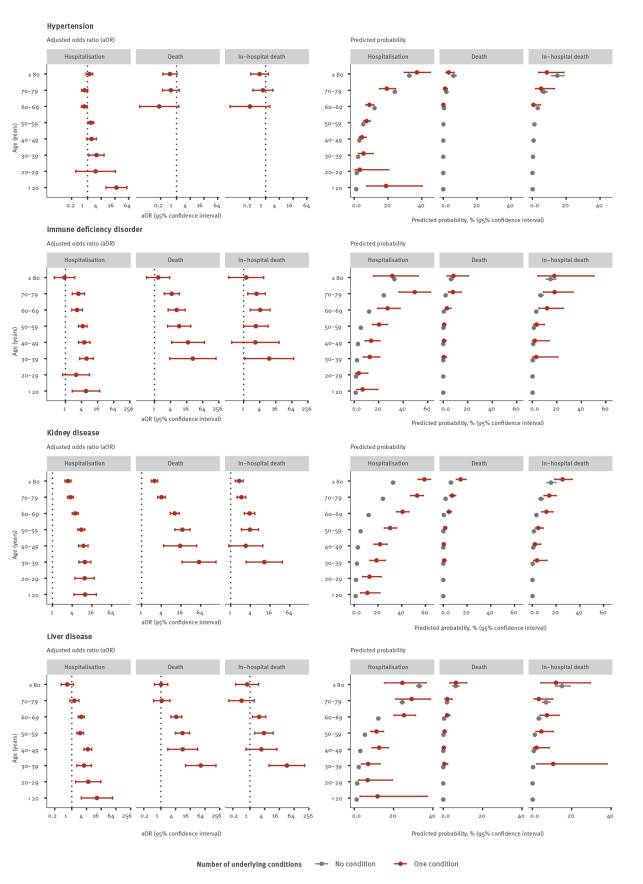
Adjusted odds ratios and predicted probabilities of hospitalisation, death and in-hospital death for the second set of underlying condition compared with COVID-19 cases without an underlying condition, EU/EEA^a^, 2 June–13 December 2020^b^

**Figure 3:**
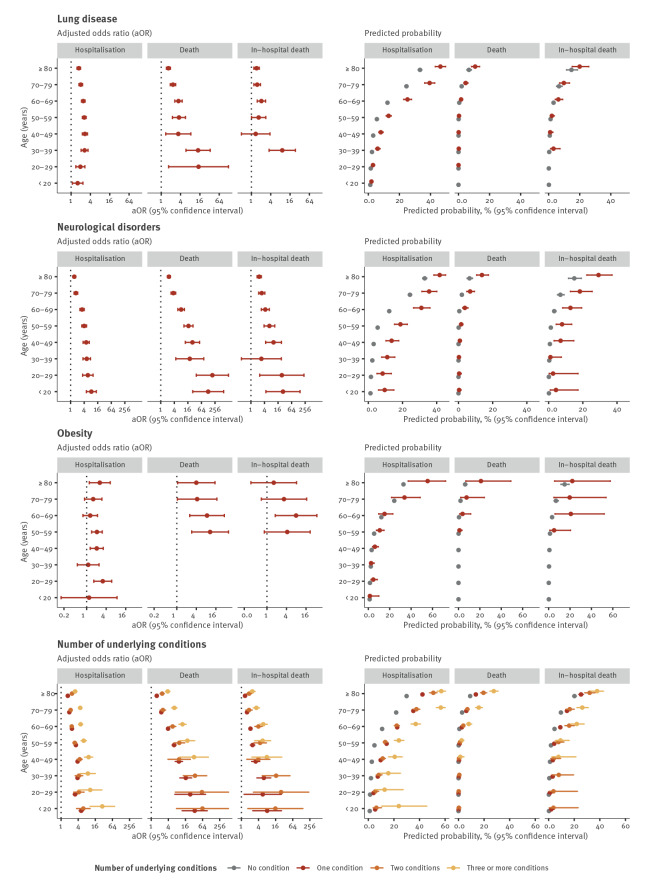
Adjusted odds ratios and predicted probabilities of hospitalisation, death and in-hospital death for the third set of underlying condition compared with COVID-19 cases without an underlying condition, EU/EEA^a^, 2 June–13 December 2020^b^

Age was a significant effect modifier in 29 of 39 age interaction models. Only in the models for asthma (all outcomes), immune deficiency disorder (fatal outcomes), hypertension (fatal outcomes) and obesity (all outcomes) were no age interactions found (p > 0.05). Notably for cancer, cardiac disorder, diabetes, kidney disease, liver disease and neurological disorders, the aOR for the association between the exposure and the outcome decreased with age (Table 2, Table 3, Table 4). Conversely, predicted probabilities of all outcomes increased with age. Additional probability of the outcome was conferred to cases of the same age with an underlying condition for many of the exposures. For some conditions, the probability of the outcome was at least as high in younger individuals with the condition as older cases without the condition, such as hospitalisation in people with cardiac disease or neurological disorders aged 50–59 years compared with those aged 60–69 years without these conditions (Figure 1, Figure 2, Figure 3).

COVID-19: coronavirus disease; EU/EEA: European Union/European Economic Area.

^a^ Countries included in this analysis: Czechia, Finland, Ireland, Italy, Luxembourg, Malta, Norway, Poland and Slovakia.

^b^ The underlying numbers for these graphs can be found in the Supplementary material.

COVID-19: coronavirus disease; EU/EEA: European Union/European Economic Area.

^a^ Countries included in this analysis: Czechia, Finland, Ireland, Italy, Luxembourg, Malta, Norway, Poland and Slovakia.

^b^ The underlying numbers for these graphs can be found in the Supplementary material.

COVID-19: coronavirus disease; EU/EEA: European Union/European Economic Area.

^a^ Countries included in this analysis: Czechia, Finland, Ireland, Italy, Luxembourg, Malta, Norway, Poland and Slovakia.

^b^ The underlying numbers for these graphs can be found in the Supplementary material.

For a given condition and age group, predicted probabilities of death were lower than those of hospitalisation or in-hospital death. Despite these differences in effect size, associations with death and in-hospital death were consistent in their direction and distribution across age groups (Table 2, Table 3, Table 4, Figure 1, Figure 2, Figure 3).

The presence of any (at least one) underlying condition was associated with increased odds of all outcomes in all age groups. Effect sizes increased with the number of conditions reported for all outcomes and in all age groups (Table 2, Table 3, Table 4, Figure 1, Figure 2, Figure 3).

### Age-specific associations with hospitalisation

Compared with cases of the same age with no underlying conditions, the adjusted odds of hospitalisation were higher among cases with cancer, cardiac disorder, diabetes, kidney disease, lung disease and neuromuscular disorder, any condition and any number of conditions in all age groups. Generally, the odds of hospitalisation decreased with increasing age. For instance, individuals aged 20–29 years with cancer were around six times (aOR: 6.20; 95% confidence interval (CI): 3.93–9.77) and people aged 80 years or older 1.4 times (95% CI: 1.33–1.53) as likely to be hospitalised than people individuals of the same age with no condition (Table 2, Figure 1, Figure 2, Figure 3).Conversely, predicted probabilities followed an increasing trend with age. In the case of cancer, the probability of hospitalisation was around 10% for cases aged 20–29 years and 40% for the oldest age group (Figure 1, Figure 2, Figure 3).

Although age did not significantly modify the association between hospitalisation and asthma or obesity (interaction p value > 0.05), significant associations for asthma were observed for cases aged 30–59 years and for obesity for cases aged 20–29, 40–59 and 80 years or older (Table 2).

### Age-specific associations with death

Compared with cases of the same age with no underlying conditions, adjusted odds of death were significantly higher among cases with cancer, cardiac disease, lung disease, diabetes, kidney disease, neuromuscular disease, any condition and number of conditions in all age groups with deaths reported. For liver disease, the association with death was not significant in individuals aged 70 years and above. For many conditions, no deaths were reported in younger age groups. Adjusted odds of death in cases aged 60–69 years were 10 times higher (aOR: 10.30; 95% CI: 7.48–14.19) in those with kidney disease than those without. This fell to less than three times higher (aOR: 2.48; 95% CI: 1.98–3.10) for those aged 80 years and above (Table 3, Figure 1, Figure 2, Figure 3). At the same time, the predicted probability of death for individuals with kidney disease increased from around 5% to 15% in cases aged 60–69 years to those aged 80 years and above (Figure 1, Figure 2, Figure 3).

Although variations exist, the predicted probability of dying remained for the most part below 10% (Figure 1, Figure 2, Figure 3). The highest probability was found in individuals aged ≥ 80 years with three or more conditions (27.56%; 95% CI: 24.37–30.99), three times higher than in individuals of the same age with no condition (9.12%; 95% CI: 8.51–9.76) (Figure 1, Figure 2, Figure 3).

Age did not significantly modify the association between obesity or immune deficiency disorder and death, although age-specific aOR for these conditions were elevated for all age groups in which deaths were reported (Table 3, Figure 1, Figure 2, Figure 3).

### Age-specific associations with in-hospital death

Trends for in-hospital death were very similar to those of death among all cases, although for in-hospital death, aOR for a given condition tended to be lower. Sample sizes for in-hospital death were smaller and CI generally larger. While cases aged 70–79 and 80 years and above had about the same relative risk of in-hospital death (ca 1.5), the absolute risk was significantly different with around 10% and 20%, respectively (Figure 1, Figure 2, Figure 3). Age-specific associations for certain conditions which were significant for the outcome death were not significant for in-hospital death (e.g. cancer and lung disease) (Table 4, Figure 1, Figure 2, Figure 3).

### Sensitivity analysis

Findings from the sensitivity analysis were generally consistent with those of the primary analysis in terms of the overall age-specific trends and direction of effects, leading to similar conclusions. Generally, effect sizes were larger in the sensitivity analysis, although some age-specific differences existed for certain conditions, such as effects in younger ages groups and larger effects in older age groups for the model with cancer and death (Supplementary Tables S9-S15 and Supplementary Figure S1 show the results of the sensitivity analysis).

## Discussion

After controlling for age, sex, reporting period and reporting country in the age-adjusted models, cases with cancer, cardiac disorder, diabetes, immune deficiency disorder, kidney disease, liver disease, lung disease, neurological disorders, obesity or any underlying condition were between 1.5 and 5.6 times as likely to be hospitalised or die than cases with no underlying condition. Asthma was associated with increased overall risk of hospitalisation, not death. Age was an important modifier of these associations, except for asthma (all outcomes), immune deficiency disorder (fatal outcomes) and obesity (all outcomes). Age-specific aOR in the age-interaction models were lower in the older age groups than in the younger age groups, whereas the opposite effect was seen for the predicted probabilities of the outcome. For many conditions, the probability of the outcome was higher among cases of the same age with an underlying condition than those without. In some instances, this difference was so large that probabilities were at least as high in younger individuals with the condition as in older cases without the condition. A ‘dose-response’ relationship was observed among multimorbid cases; i.e. effect sizes increased with number of conditions reported for all outcomes and in all age groups.

This study investigated the association between underlying conditions and severe COVID-19 in ways that were novel. Firstly, we sought to identify the individual contribution of each underlying condition by comparing cases reporting only one condition with those reporting none. Secondly, we used two fatal outcomes, allowing comparison between death among all cases and in-hospital death. Thirdly, we present estimates of both relative risk (aOR) and predicted probabilities, stratified by age. A lack of published studies employing these approaches makes direct comparison of our findings challenging. Age-specific estimates have been reported in studies focused on adolescents, which was not the focus of this study [14,15]. Nonetheless, the conditions identified as risk factors in this study are broadly in line with previous literature, and our findings build on existing evidence by identifying individual conditions that may independently increase the risk of severe outcome [3,16,17].

We found that cases with hypertension had reduced odds of fatal outcomes, in contrast to previous studies [3,16]. However, hypertension appears to be under-reported in our dataset, so the small number of deaths reported among cases with hypertension and the lack of association within any of the age strata suggest that this finding should be interpreted with caution.

The ‘dose-response’ relationship observed in this study is consistent with previous literature showing that an increased comorbidity score is associated with an estimated increased absolute risk of severe outcome and death in different age groups [18,19].

Interpretation of the results in this study is facilitated by considering together the estimates of relative risk (aOR) and predicted probabilities. The aORs for the different conditions and across different age groups tended to be higher for death than hospitalisation, but the predicted probability of hospitalisation was consistently higher for hospitalisation than for death. This suggests that underlying conditions are of greater relative importance for fatal outcomes but of greater absolute importance for hospitalisation. Similar trends were observed for death and in-hospital death. Predicted probability was higher for in-hospital death, which is expected given that this is based on a subset more severe (hospitalised) cases.

The presence of underlying conditions tended to have a larger relative effect in young than in old people, but the predicted probability of being hospitalised or dying increased with age. An interpretation for this age gradient in relative risk is that most of the risk of severe outcome among older cases was due to their age, with a small additional relative risk due to an underlying condition in older cases. Age has been shown previously to be the strongest predictor of severe COVID-19 [20,21], and the age trend in predicted probabilities among the reference groups in our study confirms this. Our estimates of predicted probability are of particular importance for intervention strategies. We show that the presence of an underlying condition can confer an additional risk among cases of the same age, with particular importance for younger age groups. Furthermore, we indicate that for some conditions, a younger person may have the same or even higher probability of severe outcome than an older person without it. The ages at which this was observed varied for different conditions, but the existence of this phenomenon in younger age groups has relevance for age and risk-factor based prioritisation of vaccination, particularly in the young.

The limitations of this study and the steps taken to address them should be considered when interpreting the results. Firstly, this study relied entirely on case-based surveillance data. Surveillance systems can vary between and within countries, leading to differences in the completeness of variables and in the accuracy of the recorded information. Most cases excluded from our analysis had missing information on underlying conditions and the proportion of hospitalised or fatal cases was slightly higher among the cases used in the analysis than in the excluded cases. This suggests that reporting of risk factor information was more complete among cases with severe disease, which may have inflated the crude risks of these outcomes in the study. Importantly, there was no difference in the age or sex distribution of included and excluded cases, which if present could have been a large potential source of bias. Secondly, the way in which age was treated in our analysis is important to consider. One objective of the study was to see if associations between underlying conditions and severe outcomes of COVID-19 varied by age. The results show that for many conditions, there is a strong age interaction in these associations, and the similarity between the age-specific patterns for the same condition across different outcomes strengthens the robustness of these findings. Information on underlying condition is reported according to a coded value list in TESSy and not to International Classification of Diseases (ICD) codes, leading to likely misclassification in the coding of the reported underlying conditions. Assuming that coding errors were non-differential, this would bias estimates towards no effect. However, the lack of ICD coding has potential implications for interpretation or comparison across age groups, since many of the terms used are non-specific, such as ‘cancer’, or could refer to different diseases or clinical manifestations in adults and children, such as ‘diabetes’. There may have been residual confounding or interactions between age and other factors that we were unable to control and that could have influenced the age-specific findings; by adjusting for age groups rather than continuous age, it is possible that there was some residual uncontrolled non-linear age effect. However, we believe that our choice of granular age groups should have minimised this issue and produced results that are more meaningful for policymakers. Because few cases younger than 20 years experienced severe outcomes and/or had underlying conditions, we were not able to adapt the age groups used in this study to the age groups used for vaccination prioritisation in younger people, since this would have reduced power to detect differences reliably and introduced instability when fitting multiple logistic regression models with small or zero cell counts. Thirdly, reverse causality may exist between underlying conditions and hospitalisation, with individuals being more likely to be hospitalised because of their underlying conditions. Changes in testing strategies over time may change the severity mix of cases, particularly in the ‘all cases’ population considered for the outcomes hospitalisation and death. For this reason, we included in-hospital deaths as an outcome, since the threshold for admission should be less prone to change over time or between countries than the chance of becoming a case. We observed consistent age trends across all three outcomes, giving us confidence that our results for hospitalisation and death among all cases are robust. This consistency across multiple outcomes also gives us some confidence that significant results were not due to chance, which is a risk in analyses that include running multiple different models. Although we consider it a strength to have limited our analysis to individuals with one condition only, we recognise that for certain underlying conditions that are commonly found together, this separation may be artificial. A useful extension of this work would therefore be to investigate associations between specific groups of conditions and severe disease. Investigation of ICU admission or length of stay as outcomes would also be desirable. We considered this but reporting of these outcomes was too incomplete in TESSy for inclusion in this study. Although smoking status is a risk factor for severe COVID-19 [22], we chose to exclude it as an exposure since data were not collected for other health behaviours and comparison with population smoking prevalence revealed it to be grossly under-reported in TESSy.

Finally, this study included data from nine EU/EEA countries, and we cannot assume that the findings are generalisable to the whole of Europe or other parts of the world where the prevalence of underlying conditions or population age structure may differ.

## Conclusion

This study showed that several underlying conditions are associated with severe COVID-19, confirmed the importance of age as the main risk factor for hospitalisation and death, and demonstrated that age is an important effect modifier in these associations. For many of the underlying conditions, their presence place cases of COVID-19 at an additional risk of hospitalisation or death compared with otherwise healthy cases who are of the same age, or even older. These findings provide new evidence that could inform a more nuanced approach to COVID-19 vaccine prioritisation that may include specific age cut-offs for individual underlying conditions. This is particularly relevant as countries consider expanding vaccination to young people, and implement age-specific dosing intervals and targeting of booster doses, in response to SARS-CoV-2 variants of concern with vaccine escape properties.

## Supplementary Data

1516000 Supplementary Material
