# Supplementary material for: Age-specific associations between underlying health conditions and hospitalisation, death and in-hospital death among confirmed COVID-19 cases: a multi-country study based on surveillance data, June to December 2020
Source: Euro Surveill. 2022 Sep 1;27(35):2100883. doi: 10.2807/1560-7917.ES.2022.27.35.2100883 (PMC9438397; doi:10.2807/1560-7917.ES.2022.27.35.2100883)
Supplement: Supplementary Material [file 21-00883_BUNDLE_Supplement.pdf]

Supplementary material

This supplementary material is hosted by Eurosurveillance as supporting information alongside the article "Age specific associations between underlying health conditions and hospitalisation, death and in-hospital death among confirmed COVID-19 cases: a multi-country study", on behalf of the authors, who remain responsible for the accuracy and appropriateness of the content. The same standards for ethics, copyright, attributions and permissions as for the article apply. Supplements are not edited by Eurosurveillance and the journal is not responsible for the maintenance of any links or email addresses provided therein.

Supplementary files:

Table S1 Characteristics of COVID-19 cases included in the primary analysis 3

Table S2 Distribution of outcome and exposure variables across all cases, cases dropped from the analysis due to missing information and those included in the analysis 4

Table S3 Number of cases, total and with outcome, by age group included in the age-interaction models for hospitalisation....... 5

Table S4 Number of cases, total and with outcome, by age group included in the age-interaction models for death6

Table S5 Number of cases, total and with outcome, by age group included in the age-interaction models for in-hospital deaths 7

Table S6 Predicted probabilities of hospitalisation for each underlying conditions and exposure compared to COVID-19 cases without an underlying condition, 2 June – 13 December 2020 8

Table S7 Predicted probabilities of death for each underlying conditions and exposure compared to COVID-19 cases without an underlying condition, 2 June – 13 December 2020....... 10

Table S8 Predicted probabilities of in-hospital death for each underlying conditions and exposure compared to COVID-19 cases without an underlying condition, 2 June – 13 December 2020 12

Table S9 Distribution of study population by age and sex with crude attack rates (AR) for each underlying condition and outcome for the sensitivity analysis, 6 February – 13 December, 2020 14

Table S10 Age-adjusted and age-stratum specific associations between underlying condition and hospitalisation for the sensitivity analysis, 6 February – 13 December 2020....... 15

Table S11 Age-adjusted and age-stratum specific associations between underlying condition and death for the sensitivity analysis, 6 February – 13 December 2020 16

Table S12 Age-adjusted and age-stratum specific associations between underlying condition and in-hospital death for the sensitivity analysis, 6 February – 13 December 2020....... 17

Table S13 Predicted probabilities of hospitalisation for each underlying conditions and exposure compared to COVID-19 cases without an underlying condition for the sensitivity analysis, 7 February – 13 December 2020....... 18

Table S14 Predicted probabilities of death for each underlying conditions and exposure compared to COVID-19 cases without an underlying condition for the sensitivity analysis, 7 February – 13 December 2020 20

Table S15 Predicted probabilities of in-hospital death for each underlying conditions and exposure compared to COVID-19 cases without an underlying condition for the sensitivity analysis, 7 February – 13 December 2020....... 22

Figure S1 Predicted probabilities of hospitalisation, death and in-hospital death for each underlying conditions compared to COVID-19 cases without an underlying condition for the sensitivity analysis, 7 February – 13 December 2020..24

Table S1 Characteristics of COVID-19 cases included in the primary analysis

	Cases included in analysis[1]	Hospitalisation, n (AR%)	Death, n (AR%)	In-hospital death, n (AR%)
**Sex**				
Female	396,750	31,797 (8.0)	8,623 (2.2)	6,166 (19.4)
Male	366,924	39,858 (10.9)	11,197 (3.1)	9,144 (22.9)
**Age (years)**				
<20	91,305	1,236 (1.4)	16 (0.0)	13 (1.1)
20-29	97,674	1,610 (1.6)	11 (0.0)	8 (0.5)
30-39	105,350	2,811 (2.7)	73 (0.1)	56 (2.0)
40-49	129,637	4,717 (3.6)	191 (0.1)	161 (3.4)
50-59	133,060	9,106 (6.8)	654 (0.5)	556 (6.1)
60-69	84,126	12,923 (15.4)	2,091 (2.5)	1,815 (14.0)
70-79	59,063	16,910 (28.6)	4,744 (8.0)	3,948 (23.3)
≥80	63,459	22,342 (35.2)	12,040 (19.0)	8,753 (39.2)
**Reporting period**				
June - September	73,170	10,178 (13.9)	1,294 (1.8)	1,128 (11.1)
October - December	690,504	61,477 (8.9)	18,526 (2.7)	14,182 (23.1)
**Reporting country**				
Czechia	14,204	1,315 (9.3)	83 (0.6)	82 (6.2)
Finland	4,091	584 (14.3)	90 (2.2)	79 (13.5)
Ireland	37,888	1,802 (4.8)	334 (0.9)	213 (11.8)
Italy	467,916	50,612 (10.8)	16,484 (3.5)	12,724 (25.1)
Luxembourg	7,135	384 (5.4)	52 (0.7)	46 (12.0)
Malta	10,659	694 (6.5)	202 (1.9)	145 (20.9)
Norway	10,044	305 (3.0)	117 (1.2)	60 (19.7)
Poland	97,162	10,641 (11.0)	1,360 (1.4)	1,250 (11.7)
Slovakia	114,575	5,318 (4.6)	1,098 (1.0)	711 (13.4)

[1]includes cases with and without underlying conditions, AR: attack rate

Table S2 Distribution of outcome and exposure variables across all cases, cases dropped from the analysis due to missing information and those included in the analysis

	All cases reported to TESSy[1] (n = 2,614,881)			Cases dropped due missing information (n = 1,851,207)			Cases included in analysis (n = 763,674)	
	n	%, including unknown	%, excluding unknown	n	%, including unknown	%, excluding unknown	n	%
**Hospitalisation**								
No	2,051,771	78.5	92.2	1,359,752	73.5	93.1	692,019	90.6
Yes	173,199	6.6	7.8	101,544	5.5	6.9	71,655	9.4
Unknown	389,911	14.9	NA	389,911	21.1	NA	NA	NA
**Death**								
No	2,152,145	82.3	98.0	1,408,291	76.1	98.3	743,854	97.4
Yes	44,161	1.7	2.0	24,341	1.3	1.7	19,820	2.6
Unknown	418,575	16.0	NA	418,575	22.6	NA	NA	NA
**Underlying Condition**								
No	691,883	26.5	83.1	53,670	2.9	78.1	638,213	83.6
Yes	140,515	5.4	16.9	15,054	0.8	21.9	125,461	16.4
Unknown	1,782,483	68.2	NA	1,782,483	96.3	NA	NA	NA
**Sex**								
Female	1,346,213	51.5	51.5	949,463	51.3	51.3	396,750	52.0
Male	1,268,007	48.5	48.5	901,083	48.7	48.7	366,924	48.0
Unknown	661	0.0	NA	661	0.0	NA	NA	NA
**Age (years)**								
<20	346,225	13.2	13.2	254,920	13.8	13.8	91,305	12.0
20-29	350,068	13.4	13.4	252,394	13.6	13.6	97,674	12.8
30-39	367,769	14.1	14.1	262,419	14.2	14.2	105,350	13.8
40-49	462,395	17.7	17.7	332,758	18.0	18.0	129,637	17.0
50-59	453,402	17.3	17.3	320,342	17.3	17.3	133,060	17.4
60-69	273,421	10.5	10.5	189,295	10.2	10.2	84,126	11.0
70-79	184,000	7.0	7.0	124,937	6.7	6.8	59,063	7.7
≥80	176,813	6.8	6.8	113,354	6.1	6.1	63,459	8.3
Unknown	788	0.0	NA	788	0.0	NA	NA	NA
**Reporting period**								
June - September	198,798	7.6	7.6	125,628	6.8	6.8	73,170	9.6
October - December	2,416,083	92.4	92.4	1,725,579	93.2	93.2	690,504	90.4
**Reporting country**								
Czechia	549,539	21.0	21.0	535,335	28.9	28.9	14,204	1.9
Finland	23,911	0.9	0.9	19,820	1.1	1.1	4,091	0.5
Ireland	51,423	2.0	2.0	13,535	0.7	0.7	37,888	5.0
Italy	1,643,509	62.9	62.9	1,175,593	63.5	63.5	467,916	61.3
Luxembourg	20,779	0.8	0.8	13,644	0.7	0.7	7,135	0.9
Malta	10,823	0.4	0.4	164	0.0	0.0	10,659	1.4
Norway	32,985	1.3	1.3	22,941	1.2	1.2	10,044	1.3
Poland	130,402	5.0	5.0	33,240	1.8	1.8	97,162	12.7
Slovakia	151,510	5.8	5.8	36,935	2.0	2.0	114,575	15.0

[1]TESSy: The European Surveillance System, NA: Not available

Table S3 Number of cases, total and with outcome, by age group included in the age-interaction models for hospitalisation

	**Age group (years)**							
	**<20 years**	**20-29**	**30-39**	**40-49**	**50-59**	**60-69**	**70-79**	**≥80**
	N (n)	N (n)	N (n)	N (n)	N (n)	N (n)	N (n)	N (n)
No underlying condition (reference)	87,707 (1,031)	92,087 (1,375)	97,849 (2,247)	116,970 (3,409)	110,629 (5,619)	60,411 (6,942)	36,094 (8,120)	36,466 (10,607)
Asthma	231 (6)	293 (5)	290 (12)	310 (18)	225 (24)	117 (15)	41 (12)	18 (8)
Cancer	162 (49)	235 (21)	561 (55)	1,519 (146)	3,395 (449)	3,731 (917)	4,212 (1,494)	4,019 (1,582)
Cardiac disorder	323 (22)	317 (22)	755 (83)	2,789 (324)	7,431 (1,197)	8,514 (2,035)	8,003 (3,016)	11,527 (5,200)
Diabetes	260 (25)	395 (35)	1,095 (127)	1,926 (290)	3,520 (785)	5,000 (1,477)	5,584 (2,254)	6,215 (2,620)
Hypertension	29 (4)	37 (1)	145 (7)	487 (18)	866 (54)	671 (53)	331 (52)	180 (52)
Immune deficiency disorder	45 (3)	82 (3)	108 (13)	143 (18)	193 (36)	94 (25)	59 (28)	25 (7)
Kidney disease	69 (7)	70 (10)	136 (29)	215 (48)	323 (101)	375 (157)	369 (197)	365 (210)
Liver disease	19 (2)	41 (3)	125 (9)	270 (34)	389 (44)	262 (64)	108 (30)	69 (15)
Lung disease	1,457 (28)	1,373 (39)	1,212 (69)	1,794 (129)	2,048 (249)	1,385 (332)	1,039 (392)	1,050 (458)
Neurological disorders	176 (17)	188 (15)	288 (30)	366 (46)	463 (82)	389 (118)	520 (174)	980 (365)
Obesity	71 (1)	209 (11)	277 (7)	351 (22)	355 (37)	146 (19)	60 (15)	31 (14)
Any underlying condition (≥1)	3,598 (205)	5,587 (235)	7,501 (564)	12,667 (1,308)	22,431 (3,487)	23,715 (5,981)	22,969 (8,790)	26,993 (11,735)
Number of underlying conditions (1)	3,272 (186)	5,002 (206)	6,701 (504)	11,297 (1,151)	20,171 (3,147)	21,245 (5,322)	20,684 (7,814)	24,818 (10,676)
Number of underlying conditions (2)	299 (14)	541 (24)	706 (47)	1,120 (112)	1,703 (216)	1,746 (397)	1,447 (534)	1,279 (608)
Number of underlying conditions (≥3)	27 (5)	44 (5)	94 (13)	250 (45)	557 (124)	724 (262)	838 (442)	896 (451)

N: number of cases included in the analysis; n: number of outcomes (hospitalisations)

Table S4 Number of cases, total and with outcome, by age group included in the age-interaction models for death

	Age group (years)							
	**<20 years**	**20-29**	**30-39**	**40-49**	**50-59**	**60-69**	**70-79**	**≥80**
	N (n)	N (n)	N (n)	N (n)	N (n)	N (n)	N (n)	N (n)
No underlying condition (reference)	87,707 (7)	92,087 (5)	97,849 (35)	116,970 (99)	110,629 (285)	60,411 (812)	36,094 (1,966)	36,466 (5,733)
Asthma	231 (0)	293 (0)	290 (0)	310 (0)	225 (0)	117 (2)	41 (2)	18 (1)
Cancer	162 (6)	235 (1)	561 (7)	1,519 (24)	3,395 (76)	3,731 (264)	4,212 (521)	4,019 (854)
Cardiac disorder	323 (0)	317 (1)	755 (7)	2,789 (20)	7,431 (97)	8,514 (384)	8,003 (915)	11,527 (2,832)
Diabetes	260 (0)	395 (0)	1,095 (3)	1,926 (17)	3,520 (89)	5,000 (310)	5,584 (787)	6,215 (1,457)
Hypertension	29 (0)	37 (0)	145 (0)	487 (0)	866 (0)	671 (1)	331 (6)	180 (9)
Immune deficiency disorder	45 (0)	82 (0)	108 (1)	143 (2)	193 (4)	94 (8)	59 (12)	25 (5)
Kidney disease	69 (0)	70 (0)	136 (3)	215 (3)	323 (15)	375 (46)	369 (70)	365 (119)
Liver disease	19 (0)	41 (0)	125 (2)	270 (2)	389 (9)	262 (16)	108 (7)	69 (12)
Lung disease	1,457 (0)	1,373 (1)	1,212 (6)	1,794 (5)	2,048 (19)	1,385 (62)	1,039 (127)	1,050 (263)
Neurological disorders	176 (2)	188 (2)	288 (2)	366 (8)	463 (20)	389 (40)	520 (88)	980 (278)
Obesity	71 (0)	209 (0)	277 (0)	351 (0)	355 (3)	146 (4)	60 (3)	31 (4)
Any underlying condition (≥1)	3,598 (9)	5,587 (6)	7,501 (38)	12,667 (92)	22,431 (369)	23,715 (1,279)	22,969 (2,778)	26,993 (6,307)
Number of underlying conditions (1)	3,272 (8)	5,002 (5)	6,701 (33)	11,297 (84)	20,171 (334)	21,245 (1,150)	20,684 (2,553)	24,818 (5,867)
Number of underlying conditions (2)	299 (1)	541 (1)	706 (5)	1,120 (5)	1,703 (23)	1,746 (72)	1,447 (102)	1,279 (226)
Number of underlying conditions (≥3)	27 (0)	44 (0)	94 (0)	250 (3)	557 (12)	724 (57)	838 (123)	896 (214)

N: number of cases included in the analysis; n: number of outcomes (death)

Table S5 Number of cases, total and with outcome, by age group included in the age-interaction models for in-hospital deaths

	Age group (years)							
	**<20 years**	**20-29**	**30-39**	**40-49**	**50-59**	**60-69**	**70-79**	**≥80**
	N (n)	N (n)	N (n)	N (n)	N (n)	N (n)	N (n)	N (n)
No underlying condition (reference)	1,031 (5)	1,375 (4)	2,247 (22)	3,409 (74)	5,619 (223)	6,942 (669)	8,120 (1,539)	10,607 (3,774)
Asthma	6 (0)	5 (0)	12 (0)	18 (0)	24 (0)	15 (2)	12 (1)	8 (1)
Cancer	49 (5)	21 (1)	55 (6)	146 (23)	449 (68)	917 (214)	1,494 (431)	1,582 (629)
Cardiac disorder	22 (0)	22 (1)	83 (6)	324 (19)	1,197 (92)	2,035 (356)	3,016 (824)	5,200 (2,315)
Diabetes	25 (0)	35 (0)	127 (3)	290 (16)	785 (83)	1,477 (279)	2,254 (687)	2,620 (1,145)
Hypertension	4 (0)	1 (0)	7 (0)	18 (0)	54 (0)	53 (1)	52 (5)	52 (7)
Immune deficiency disorder	3 (0)	3 (0)	13 (1)	18 (1)	36 (4)	25 (8)	28 (12)	7 (3)
Kidney disease	7 (0)	10 (0)	29 (3)	48 (3)	101 (13)	157 (42)	197 (64)	210 (105)
Liver disease	2 (0)	3 (0)	9 (2)	34 (2)	44 (6)	64 (13)	30 (3)	15 (5)
Lung disease	28 (0)	39 (0)	69 (6)	129 (4)	249 (17)	332 (58)	392 (103)	458 (204)
Neurological disorders	17 (2)	15 (1)	30 (1)	46 (8)	82 (18)	118 (36)	174 (69)	365 (197)
Obesity	1 (0)	11 (0)	7 (0)	22 (0)	37 (2)	19 (4)	15 (3)	14 (3)
Any underlying condition (≥1)	205 (8)	235 (4)	564 (34)	1,308 (87)	3,487 (333)	5,981 (1,146)	8,790 (2,409)	11,735 (4,979)
Number of underlying conditions (1)	186 (7)	206 (3)	504 (30)	1,151 (79)	3,147 (304)	5,322 (1,025)	7,814 (2,215)	10,676 (4,647)
Number of underlying conditions (2)	14 (1)	24 (1)	47 (4)	112 (5)	216 (18)	397 (67)	534 (87)	608 (183)
Number of underlying conditions (≥3)	5 (0)	5 (0)	13 (0)	45 (3)	124 (11)	262 (54)	442 (107)	451 (149)

N: number of cases included in the analysis; n: number of outcomes (in-hospital death)

Table S6 Predicted probabilities of hospitalisation for each underlying conditions and exposure compared to COVID-19 cases without an underlying condition, 2 June – 13 December 2020

	Variable value*	Age group, PP% (CI)							
		<20 years	20-29	30-39	40-49	50-59	60-69	70-79	≥80
Asthma	0	1.22 (1.13-1.31)	1.50 (1.40-1.60)	2.27 (2.14-2.42)	3.02 (2.86-3.19)	5.30 (5.04-5.58)	11.92 (11.37-12.50)	23.83 (22.86-24.82)	32.35 (31.18-33.53)
Asthma	1	2.39 (1.06-5.27)	1.75 (0.72-4.16)	4.35 (2.46-7.57)	6.48 (4.09-10.14)	11.70 (7.86-17.06)	14.50 (8.88-22.79)	34.39 (20.68-51.31)	48.23 (26.40-70.76)
Cancer	0	1.25 (1.16-1.35)	1.54 (1.44-1.65)	2.34 (2.20-2.48)	3.11 (2.94-3.28)	5.46 (5.19-5.74)	12.23 (11.67-12.81)	24.39 (23.43-25.39)	33.12 (31.96-34.31)
Cancer	1	31.84 (24.87-39.74)	8.84 (5.80-13.25)	10.33 (7.97-13.28)	10.75 (9.15-12.58)	14.70 (13.36-16.14)	25.62 (23.96-27.36)	36.42 (34.60-38.29)	41.37 (39.43-43.33)
Cardiac disorder	0	1.21 (1.12-1.30)	1.49 (1.39-1.59)	2.27 (2.14-2.41)	3.01 (2.85-3.17)	5.27 (5.02-5.54)	11.84 (11.31-12.38)	23.64 (22.73-24.58)	32.16 (31.05-33.28)
Cardiac disorder	1	6.26 (4.13-9.38)	6.21 (4.09-9.30)	9.92 (8.01-12.22)	10.45 (9.34-11.68)	14.81 (13.86-15.80)	22.14 (21.00-23.33)	36.49 (35.01-38.00)	46.75 (45.28-48.22)
Diabetes	0	1.17 (1.09-1.26)	1.44 (1.35-1.54)	2.19 (2.06-2.32)	2.91 (2.75-3.07)	5.10 (4.85-5.37)	11.49 (10.97-12.03)	23.06 (22.15-24.01)	31.44 (30.33-32.58)
Diabetes	1	9.39 (6.38-13.60)	8.63 (6.23-11.85)	12.13 (10.23-14.33)	14.34 (12.77-16.07)	20.60 (19.13-22.14)	27.85 (26.33-29.43)	39.69 (37.98-41.42)	43.50 (41.79-45.22)
Hypertension	0	1.23 (1.14-1.32)	1.51 (1.41-1.62)	2.30 (2.17-2.44)	3.06 (2.89-3.23)	5.36 (5.10-5.64)	12.04 (11.50-12.60)	24.03 (23.09-25.00)	32.59 (31.46-33.74)
Hypertension	1	18.85 (7.43-40.19)	3.35 (0.47-20.43)	5.52 (2.65-11.17)	4.56 (2.88-7.14)	7.36 (5.65-9.53)	9.02 (6.92-11.66)	19.25 (14.94-24.44)	37.06 (29.68-45.10)
Immune deficiency disorder	0	1.24 (1.15-1.34)	1.53 (1.43-1.64)	2.32 (2.19-2.47)	3.09 (2.92-3.27)	5.42 (5.14-5.71)	12.16 (11.59-12.75)	24.23 (23.24-25.26)	32.84 (31.64-34.06)
Immune deficiency disorder	1	6.86 (2.22-19.33)	3.74 (1.20-11.02)	12.73 (7.49-20.81)	13.89 (8.89-21.05)	20.32 (14.98-26.96)	27.30 (19.05-37.46)	49.42 (36.59-62.33)	31.10 (15.66-52.31)
Kidney disease	0	1.24 (1.15-1.34)	1.53 (1.42-1.64)	2.32 (2.18-2.47)	3.08 (2.91-3.26)	5.41 (5.13-5.69)	12.13 (11.57-12.72)	24.19 (23.21-25.21)	32.77 (31.59-33.98)
Kidney disease	1	11.11 (5.37-21.57)	12.92 (6.97-22.72)	18.80 (13.19-26.08)	21.78 (16.66-27.95)	30.31 (25.40-35.72)	40.73 (35.65-46.01)	53.02 (47.66-58.31)	59.15 (53.81-64.27)
Liver disease	0	1.25 (1.16-1.35)	1.54 (1.44-1.65)	2.34 (2.20-2.49)	3.11 (2.94-3.29)	5.46 (5.19-5.75)	12.25 (11.68-12.85)	24.41 (23.42-25.43)	33.06 (31.87-34.27)
Liver disease	1	11.90 (3.00-37.14)	6.85 (2.19-19.42)	7.08 (3.70-13.14)	12.70 (9.16-17.34)	11.32 (8.48-14.94)	25.34 (20.25-31.21)	29.22 (21.18-38.80)	24.49 (15.39-36.65)
Lung disease	0	1.26 (1.17-1.36)	1.55 (1.45-1.66)	2.36 (2.22-2.51)	3.13 (2.96-3.31)	5.50 (5.23-5.79)	12.32 (11.76-12.91)	24.54 (23.56-25.54)	33.25 (32.07-34.45)
Lung disease	1	2.01 (1.38-2.90)	2.98 (2.17-4.07)	5.97 (4.72-7.54)	8.03 (6.76-9.52)	13.04 (11.52-14.73)	25.21 (22.77-27.82)	39.44 (36.24-42.73)	46.39 (43.10-49.71)

Neurological disorders	0	1.25 (1.16-1.35)	1.54 (1.44-1.65)	2.34 (2.20-2.49)	3.11 (2.94-3.29)	5.45 (5.17-5.74)	12.22 (11.65-12.82)	24.35 (23.36-25.37)	32.98 (31.79-34.20)
Neurological disorders	1	9.56 (5.99-14.94)	8.35 (5.07-13.45)	11.08 (7.81-15.49)	13.63 (10.31-17.79)	18.79 (15.31-22.84)	31.05 (26.45-36.06)	35.58 (31.32-40.08)	41.74 (38.37-45.20)
Obesity	0	1.25 (1.15-1.34)	1.53 (1.43-1.64)	2.33 (2.19-2.47)	3.09 (2.92-3.27)	5.43 (5.16-5.72)	12.18 (11.61-12.77)	24.27 (23.29-25.28)	32.88 (31.70-34.09)
Obesity	1	1.48 (0.21-9.81)	4.66 (2.55-8.36)	2.59 (1.22-5.40)	6.14 (3.99-9.32)	10.64 (7.63-14.66)	15.16 (9.71-22.91)	33.81 (21.70-48.48)	55.45 (37.31-72.24)
Any underlying condition (≥1)	0	1.16 (1.08-1.23)	1.43 (1.35-1.51)	2.17 (2.07-2.27)	2.87 (2.76-2.99)	5.04 (4.87-5.22)	11.33 (10.98-11.70)	22.78 (22.15-23.42)	31.21 (30.46-31.97)
Any underlying condition (≥1)	1	5.69 (4.97-6.51)	4.36 (3.84-4.95)	7.76 (7.15-8.42)	10.32 (9.76-10.90)	14.99 (14.45-15.54)	23.84 (23.16-24.54)	37.29 (36.44-38.15)	44.55 (43.68-45.43)
Number of underlying conditions	0	1.10 (1.03-1.17)	1.36 (1.28-1.44)	2.06 (1.96-2.16)	2.72 (2.61-2.84)	4.78 (4.61-4.96)	10.78 (10.43-11.15)	21.76 (21.13-22.41)	29.90 (29.14-30.68)
Number of underlying conditions	1	5.36 (4.65-6.18)	4.11 (3.58-4.70)	7.38 (6.77-8.05)	9.64 (9.08-10.23)	14.26 (13.70-14.83)	22.55 (21.84-23.29)	35.17 (34.26-36.09)	42.30 (41.36-43.25)
Number of underlying conditions	2	6.28 (3.75-10.34)	5.49 (3.70-8.08)	8.20 (6.20-10.77)	11.22 (9.38-13.36)	13.19 (11.60-14.96)	22.21 (20.25-24.30)	37.60 (35.00-40.28)	50.80 (47.89-53.70)
Number of underlying conditions	≥3	23.81 (10.39-45.72)	12.71 (5.35-27.29)	15.62 (9.25-25.15)	20.62 (15.72-26.56)	23.88 (20.34-27.83)	37.00 (33.39-40.77)	56.66 (53.13-60.11)	57.02 (53.61-60.36)

*0 indicates no underlying health condition, reference group; Probabilities were estimated from the fully adjusted age-interaction models. Baseline risks may therefore vary between the different models. CI: Confidence interval; NA: Not available as no outcome occurred within the age group, PP: predicted probabilities.

Table S7 Predicted probabilities of death for each underlying conditions and exposure compared to COVID-19 cases without an underlying condition, 2 June – 13 December 2020

	Variable value*	Age group, PP% (CI)							
		<20 years	20-29	30-39	40-49	50-59	60-69	70-79	≥80
Asthma	0	0.00 (0.00-0.01)	0.00 (0.00-0.00)	0.01 (0.01-0.02)	0.03 (0.02-0.04)	0.09 (0.07-0.12)	0.47 (0.37-0.61)	1.94 (1.52-2.48)	6.40 (5.07-8.04)
Asthma	1	NA	NA	NA	NA	NA	0.79 (0.19-3.21)	3.02 (0.71-11.95)	2.93 (0.38-19.47)
Cancer	0	0.00 (0.00-0.01)	0.00 (0.00-0.01)	0.02 (0.01-0.02)	0.04 (0.03-0.05)	0.11 (0.09-0.14)	0.56 (0.45-0.69)	2.28 (1.84-2.81)	7.47 (6.12-9.08)
Cancer	1	1.44 (0.62-3.29)	0.16 (0.02-1.15)	0.52 (0.24-1.12)	0.69 (0.44-1.08)	0.95 (0.70-1.29)	2.78 (2.19-3.52)	4.76 (3.82-5.93)	9.13 (7.42-11.17)
Cardiac disorder	0	0.00 (0.00-0.01)	0.00 (0.00-0.01)	0.02 (0.01-0.02)	0.04 (0.03-0.05)	0.11 (0.09-0.13)	0.56 (0.46-0.67)	2.28 (1.91-2.73)	7.48 (6.33-8.81)
Cardiac disorder	1	NA	0.12 (0.02-0.86)	0.38 (0.18-0.82)	0.28 (0.18-0.45)	0.49 (0.37-0.63)	1.71 (1.40-2.09)	4.66 (3.89-5.57)	11.97 (10.19-14.02)
Diabetes	0	0.00 (0.00-0.01)	0.00 (0.00-0.00)	0.01 (0.01-0.02)	0.03 (0.02-0.04)	0.09 (0.07-0.11)	0.44 (0.35-0.57)	1.83 (1.44-2.32)	6.02 (4.79-7.53)
Diabetes	1	NA	NA	0.10 (0.03-0.31)	0.28 (0.16-0.47)	0.71 (0.52-0.98)	1.76 (1.35-2.28)	4.37 (3.44-5.55)	8.67 (6.90-10.83)
Hypertension	0	0.00 (0.00-0.01)	0.00 (0.00-0.00)	0.01 (0.01-0.02)	0.03 (0.02-0.04)	0.09 (0.07-0.11)	0.45 (0.35-0.58)	1.84 (1.43-2.36)	6.07 (4.78-7.69)
Hypertension	1	NA	NA	NA	NA	NA	0.08 (0.01-0.56)	1.04 (0.45-2.41)	3.21 (1.57-6.44)
Immune deficiency disorder	0	0.00 (0.00-0.01)	0.00 (0.00-0.00)	0.01 (0.01-0.02)	0.03 (0.02-0.04)	0.09 (0.07-0.12)	0.47 (0.36-0.60)	1.92 (1.49-2.46)	6.32 (4.97-7.99)
Immune deficiency disorder	1	NA	NA	0.35 (0.05-2.52)	0.55 (0.13-2.24)	0.75 (0.27-2.07)	3.06 (1.44-6.38)	7.92 (4.14-14.63)	8.43 (3.18-20.51)
Kidney disease	0	0.00 (0.00-0.01)	0.00 (0.00-0.00)	0.01 (0.01-0.02)	0.03 (0.02-0.04)	0.09 (0.07-0.12)	0.48 (0.37-0.62)	1.97 (1.55-2.50)	6.47 (5.15-8.10)
Kidney disease	1	NA	NA	0.76 (0.24-2.41)	0.48 (0.15-1.52)	1.63 (0.93-2.85)	4.74 (3.26-6.84)	7.55 (5.42-10.44)	14.64 (11.00-19.21)
Liver disease	0	0.00 (0.00-0.01)	0.00 (0.00-0.00)	0.01 (0.01-0.02)	0.03 (0.02-0.04)	0.09 (0.07-0.12)	0.47 (0.36-0.60)	1.92 (1.49-2.46)	6.31 (4.97-7.98)
Liver disease	1	NA	NA	0.56 (0.13-2.26)	0.24 (0.06-0.98)	0.69 (0.34-1.38)	1.96 (1.12-3.40)	2.09 (0.94-4.58)	6.42 (3.37-11.90)
Lung disease	0	0.00 (0.00-0.01)	0.00 (0.00-0.01)	0.01 (0.01-0.02)	0.03 (0.02-0.04)	0.10 (0.07-0.12)	0.50 (0.39-0.63)	2.04 (1.62-2.56)	6.71 (5.40-8.30)
Lung disease	1	NA	0.03 (0.00-0.22)	0.19 (0.08-0.45)	0.11 (0.04-0.27)	0.34 (0.21-0.57)	1.68 (1.20-2.35)	4.61 (3.47-6.08)	10.73 (8.42-13.59)

Neurological disorders	0	0.00 (0.00-0.01)	0.00 (0.00-0.00)	0.01 (0.01-0.02)	0.03 (0.02-0.04)	0.09 (0.07-0.12)	0.48 (0.37-0.62)	1.97 (1.53-2.54)	6.47 (5.07-8.22)
Neurological disorders	1	0.36 (0.09-1.49)	0.39 (0.09-1.59)	0.26 (0.06-1.06)	0.80 (0.38-1.67)	1.56 (0.94-2.60)	3.72 (2.48-5.54)	6.80 (4.91-9.35)	13.71 (10.60-17.56)
Obesity	0	0.00 (0.00-0.01)	0.00 (0.00-0.00)	0.01 (0.01-0.02)	0.03 (0.02-0.04)	0.09 (0.07-0.12)	0.46 (0.36-0.60)	1.89 (1.47-2.43)	6.24 (4.91-7.90)
Obesity	1	NA	NA	NA	NA	0.94 (0.27-3.24)	3.81 (1.20-11.39)	7.56 (2.06-24.18)	20.99 (6.86-48.96)
Any underlying condition (≥1)	0	0.00 (0.00-0.01)	0.00 (0.00-0.01)	0.02 (0.02-0.03)	0.05 (0.04-0.07)	0.16 (0.14-0.18)	0.82 (0.75-0.90)	3.36 (3.12-3.62)	10.79 (10.16-11.46)
Any underlying condition (≥1)	1	0.16 (0.08-0.31)	0.08 (0.04-0.18)	0.37 (0.27-0.51)	0.50 (0.40-0.62)	1.03 (0.92-1.16)	3.26 (3.02-3.52)	7.50 (7.03-8.00)	16.31 (15.44-17.21)
Number of underlying conditions	0	0.00 (0.00-0.01)	0.00 (0.00-0.01)	0.02 (0.01-0.03)	0.05 (0.04-0.06)	0.13 (0.12-0.15)	0.69 (0.62-0.76)	2.82 (2.60-3.05)	9.12 (8.51-9.76)
Number of underlying conditions	1	0.14 (0.07-0.28)	0.07 (0.03-0.16)	0.31 (0.22-0.44)	0.42 (0.34-0.53)	0.84 (0.74-0.96)	2.63 (2.41-2.88)	6.16 (5.71-6.65)	13.55 (12.68-14.47)
Number of underlying conditions	2	0.26 (0.04-1.81)	0.17 (0.02-1.21)	0.66 (0.27-1.58)	0.43 (0.18-1.04)	1.28 (0.85-1.93)	3.76 (2.98-4.75)	6.92 (5.68-8.39)	19.52 (17.19-22.09)
Number of underlying conditions	≥3	NA	NA	NA	1.42 (0.46-4.33)	2.44 (1.38-4.25)	8.01 (6.19-10.31)	15.85 (13.34-18.74)	27.56 (24.37-30.99)

*0 indicates no underlying health condition, reference group; Probabilities were estimated from the fully adjusted age-interaction models. Baseline risks may therefore vary between the different models. CI: Confidence interval; NA: Not available as no outcome occurred within the age group, PP: predicted probabilities

Table S8 Predicted probabilities of in-hospital death for each underlying conditions and exposure compared to COVID-19 cases without an underlying condition, 2 June – 13 December 2020

	Variable value*	Age group, PP% (CI)							
		**<20, years**	**20-29**	**30-39**	**40-49**	**50-59**	**60-69**	**70-79**	**≥80**
Asthma	0	0.17 (0.07-0.43)	0.11 (0.04-0.31)	0.37 (0.22-0.61)	0.75 (0.52-1.08)	1.29 (0.94-1.75)	3.28 (2.47-4.36)	6.96 (5.31-9.09)	15.20 (11.86-19.28)
Asthma	1	NA	NA	NA	NA	NA	7.30 (1.63-27.15)	3.92 (0.48-25.52)	8.04 (1.01-42.73)
Cancer	0	0.20 (0.08-0.50)	0.13 (0.05-0.36)	0.43 (0.27-0.70)	0.88 (0.63-1.22)	1.51 (1.14-1.99)	3.84 (2.99-4.92)	8.09 (6.40-10.19)	17.39 (14.08-21.30)
Cancer	1	3.94 (1.54-9.70)	1.94 (0.26-13.09)	4.67 (1.98-10.64)	6.47 (3.98-10.34)	6.15 (4.38-8.58)	9.53 (7.29-12.35)	11.88 (9.31-15.03)	18.21 (14.55-22.55)
Cardiac disorder	0	0.20 (0.08-0.49)	0.13 (0.05-0.36)	0.43 (0.27-0.69)	0.88 (0.64-1.20)	1.51 (1.18-1.94)	3.84 (3.08-4.79)	8.10 (6.60-9.92)	17.47 (14.54-20.84)
Cardiac disorder	1	NA	2.12 (0.28-14.09)	2.93 (1.26-6.67)	2.18 (1.32-3.59)	2.69 (2.00-3.61)	6.83 (5.43-8.56)	11.83 (9.64-14.43)	23.13 (19.43-27.29)
Diabetes	0	0.17 (0.07-0.42)	0.11 (0.04-0.30)	0.36 (0.22-0.59)	0.73 (0.51-1.04)	1.25 (0.93-1.69)	3.20 (2.43-4.21)	6.79 (5.23-8.78)	14.83 (11.68-18.65)
Diabetes	1	NA	NA	0.72 (0.22-2.30)	1.62 (0.92-2.84)	3.05 (2.15-4.29)	5.79 (4.34-7.67)	10.51 (8.10-13.54)	18.04 (14.21-22.63)
Hypertension	0	0.16 (0.07-0.41)	0.11 (0.04-0.30)	0.35 (0.21-0.59)	0.72 (0.50-1.04)	1.24 (0.91-1.69)	3.16 (2.37-4.21)	6.72 (5.11-8.79)	14.72 (11.45-18.72)
Hypertension	1	NA	NA	NA	NA	NA	0.67 (0.09-4.81)	5.18 (1.94-13.10)	8.56 (3.68-18.66)
Immune deficiency disorder	0	0.17 (0.07-0.42)	0.11 (0.04-0.31)	0.36 (0.22-0.60)	0.73 (0.50-1.05)	1.25 (0.91-1.72)	3.20 (2.38-4.29)	6.79 (5.13-8.95)	14.87 (11.49-19.02)
Immune deficiency disorder	1	NA	NA	3.18 (0.41-20.88)	1.99 (0.26-13.59)	3.52 (1.21-9.77)	12.07 (5.25-25.36)	18.12 (8.95-33.24)	17.98 (4.52-50.40)
Kidney disease	0	0.18 (0.07-0.45)	0.12 (0.04-0.33)	0.38 (0.23-0.63)	0.78 (0.55-1.11)	1.34 (0.99-1.81)	3.42 (2.58-4.51)	7.23 (5.55-9.37)	15.72 (12.36-19.80)
Kidney disease	1	NA	NA	3.94 (1.18-12.37)	2.24 (0.68-7.11)	5.01 (2.68-9.16)	11.82 (7.87-17.39)	14.24 (9.93-19.99)	25.43 (18.77-33.49)
Liver disease	0	0.17 (0.07-0.43)	0.11 (0.04-0.31)	0.37 (0.22-0.60)	0.74 (0.52-1.07)	1.28 (0.94-1.74)	3.26 (2.44-4.34)	6.92 (5.26-9.06)	15.13 (11.77-19.23)
Liver disease	1	NA	NA	10.61 (2.24-38.05)	2.15 (0.51-8.69)	4.59 (1.90-10.68)	7.43 (3.91-13.64)	3.26 (0.97-10.38)	12.06 (4.29-29.55)
Lung disease	0	0.16 (0.07-0.41)	0.11 (0.04-0.30)	0.35 (0.21-0.58)	0.72 (0.50-1.03)	1.23 (0.91-1.67)	3.15 (2.38-4.16)	6.69 (5.12-8.68)	14.67 (11.50-18.54)
Lung disease	1	NA	NA	3.13 (1.31-7.28)	0.97 (0.35-2.70)	2.06 (1.18-3.57)	6.25 (4.29-9.02)	9.78 (7.05-13.41)	19.96 (15.16-25.81)

Neurological disorders	0	0.17 (0.07-0.42)	0.11 (0.04-0.31)	0.36 (0.22-0.60)	0.74 (0.51-1.07)	1.27 (0.93-1.74)	3.25 (2.42-4.34)	6.88 (5.20-9.04)	15.00 (11.62-19.15)
Neurological disorders	1	4.28 (0.95-17.21)	2.58 (0.34-17.20)	1.05 (0.14-7.42)	7.00 (3.18-14.70)	7.85 (4.44-13.49)	12.76 (8.20-19.31)	18.27 (12.75-25.48)	29.18 (22.36-37.10)
Obesity	0	0.17 (0.07-0.42)	0.11 (0.04-0.31)	0.36 (0.22-0.60)	0.73 (0.51-1.06)	1.26 (0.92-1.73)	3.21 (2.39-4.30)	6.82 (5.15-8.98)	14.92 (11.54-19.09)
Obesity	1	NA	NA	NA	NA	5.10 (1.11-20.43)	20.76 (5.96-51.99)	19.68 (4.92-53.72)	22.12 (5.59-57.67)
Any underlying condition (≥1)	0	0.29 (0.12-0.69)	0.18 (0.07-0.49)	0.60 (0.39-0.92)	1.24 (0.97-1.57)	2.14 (1.84-2.48)	5.39 (4.86-5.97)	11.17 (10.28-12.12)	23.11 (21.64-24.65)
Any underlying condition (≥1)	1	2.30 (1.14-4.59)	1.20 (0.45-3.16)	3.78 (2.68-5.30)	3.91 (3.13-4.86)	5.29 (4.66-5.99)	11.21 (10.29-12.19)	16.90 (15.74-18.13)	29.18 (27.56-30.85)
Number of underlying conditions	0	0.25 (0.10-0.59)	0.16 (0.06-0.42)	0.51 (0.34-0.79)	1.05 (0.82-1.34)	1.81 (1.55-2.11)	4.59 (4.11-5.13)	9.58 (8.74-10.50)	20.17 (18.69-21.73)
Number of underlying conditions	1	1.95 (0.92-4.09)	0.87 (0.28-2.69)	3.09 (2.13-4.45)	3.27 (2.58-4.14)	4.33 (3.77-4.98)	9.11 (8.25-10.06)	14.31 (13.14-15.56)	25.30 (23.57-27.12)
Number of underlying conditions	2	3.73 (0.50-23.12)	3.76 (0.52-22.57)	7.93 (2.96-19.54)	4.01 (1.66-9.36)	7.72 (4.87-12.01)	16.05 (12.69-20.11)	16.44 (13.37-20.05)	32.05 (28.08-36.29)
Number of underlying conditions	3+	NA	NA	NA	7.68 (2.49-21.36)	9.30 (5.19-16.11)	22.00 (17.11-27.81)	26.27 (22.03-31.01)	37.87 (33.05-42.94)

*0 indicates no underlying health condition, reference group; Probabilities were estimated from the fully adjusted age-interaction models. Baseline risks may therefore slightly vary between the different models. CI: Confidence interval; NA: Not available as no outcome occurred within the age group, PP: predicted probabilities

Table S9 Distribution of study population by age and sex with crude attack rates (AR) for each underlying condition and outcome for the sensitivity analysis, 6 February – 13 December, 2020

	Population size	Sex, n (%)	Age group, n(%)								Outcome, n (AR%)		
		Male	<20	20-29	30-39	40-49	50-59	60-69	70-79	≥80	Hospitalisation	Death	In-hospital death
No underlying condition (reference)	693975	328543 (47.3)	90316 (13.0)	98341 (14.2)	105688 (15.2)	127274 (18.3)	121709 (17.5)	66430 (9.6)	40265 (5.8)	43952 (6.3)	52333 (7.5)	12084 (1.7)	8541 (16.3)
Asthma	1804	770 (42.7)	253 (14.0)	351 (19.5)	341 (18.9)	387 (21.5)	268 (14.9)	133 (7.4)	47 (2.6)	24 (1.3)	137 (7.6)	6 (0.3)	5 (3.6)
Cancer	25252	11777 (46.6)	185 (0.7)	279 (1.1)	686 (2.7)	1896 (7.5)	4360 (17.3)	4956 (19.6)	6124 (24.3)	6766 (26.8)	8785 (34.8)	3798 (15.0)	3090 (35.2)
Cardiac disorder	59390	29751 (50.1)	364 (0.6)	394 (0.7)	966 (1.6)	3634 (6.1)	9840 (16.6)	11416 (19.2)	11995 (20.2)	20781 (35.0)	22693 (38.2)	9558 (16.1)	7929 (34.9)
Diabetes	33272	17462 (52.5)	282 (0.8)	453 (1.4)	1235 (3.7)	2327 (7.0)	4458 (13.4)	6583 (19.8)	7952 (23.9)	9982 (30.0)	13038 (39.2)	5335 (16.0)	4393 (33.7)
Hypertension	3239	1538 (47.5)	31 (1.0)	47 (1.5)	175 (5.4)	589 (18.2)	1019 (31.5)	763 (23.6)	364 (11.2)	251 (7.7)	302 (9.3)	41 (1.3)	18 (6.0)
Immune deficiency disorder	1160	492 (42.4)	50 (4.3)	112 (9.7)	167 (14.4)	232 (20.0)	323 (27.8)	132 (11.4)	89 (7.7)	55 (4.7)	283 (24.4)	70 (6.0)	58 (20.5)
Kidney disease	2739	1553 (56.7)	78 (2.8)	92 (3.4)	184 (6.7)	300 (11.0)	425 (15.5)	495 (18.1)	536 (19.6)	629 (23.0)	1262 (46.1)	479 (17.5)	419 (33.2)
Liver disease	1499	831 (55.4)	22 (1.5)	50 (3.3)	141 (9.4)	313 (20.9)	433 (28.9)	311 (20.7)	132 (8.8)	97 (6.5)	284 (18.9)	75 (5.0)	54 (19.0)
Lung disease	14815	7043 (47.5)	1561 (10.5)	1680 (11.3)	1552 (10.5)	2323 (15.7)	2656 (17.9)	1820 (12.3)	1514 (10.2)	1709 (11.5)	2911 (19.6)	987 (6.7)	788 (27.1)
Neurological disorders	4613	1880 (40.8)	182 (3.9)	219 (4.7)	348 (7.5)	452 (9.8)	580 (12.6)	516 (11.2)	772 (16.7)	1544 (33.5)	1317 (28.5)	842 (18.3)	536 (40.7)
Obesity	1635	820 (50.2)	72 (4.4)	226 (13.8)	311 (19.0)	385 (23.5)	384 (23.5)	162 (9.9)	63 (3.9)	32 (2.0)	163 (10.0)	19 (1.2)	17 (10.4)
Any underlying condition (≥1)	174379	85643 (49.1)	3898 (2.2)	6565 (3.8)	9055 (5.2)	15838 (9.1)	28738 (16.5)	31117 (17.8)	33211 (19.0)	45957 (26.4)	57504 (33.0)	23357 (13.4)	18781 (32.7)
Number of underlying conditions (1)	157591	77492 (49.2)	3541 (2.2)	5865 (3.7)	8086 (5.1)	14142 (9.0)	25840 (16.4)	27902 (17.7)	29975 (19.0)	42240 (26.8)	52053 (33.0)	21294 (13.5)	17386 (33.4)
Number of underlying conditions (2)	11647	5578 (47.9)	326 (2.8)	644 (5.5)	839 (7.2)	1367 (11.7)	2157 (18.5)	2220 (19.1)	1962 (16.8)	2132 (18.3)	3114 (26.7)	1070 (9.2)	716 (23.0)
Number of underlying conditions (≥3)	5141	2573 (50.0)	31 (0.6)	56 (1.1)	130 (2.5)	329 (6.4)	741 (14.4)	995 (19.4)	1274 (24.8)	1585 (30.8)	2337 (45.5)	993 (19.3)	679 (29.1)

Table S10 Age-adjusted and age-stratum specific associations between underlying condition and hospitalisation for the sensitivity analysis, 6 February – 13 December 2020

	Age-adjusted model		Age-interaction model, stratum-specific aOR (95% CI)								
	**aOR**	**p-value[1]**	**<20**	**20-29**	**30-39**	**40-49**	**50-59**	**60-69**	**70-79**	**≥80**	**Interaction p-value[1]**
Asthma	1.8 (1.4-2.3)	<0.0001	2.0 (0.9-4.6)	1.2 (0.5-2.9)	2.0 (1.1-3.5)	2.2 (1.4-3.6)	2.4 (1.5-3.7)	1.3 (0.7-2.2)	1.7 (0.8-3.4)	1.9 (0.7-5.1)	0.64
Cancer	2.0 (1.9-2.1)	<0.0001	36.8 (26.0-52.1)	6.2 (3.9-9.8)	4.8 (3.6-6.4)	3.8 (3.1-4.5)	3.0 (2.7-3.3)	2.5 (2.3-2.7)	1.8 (1.7-1.9)	1.4 (1.3-1.5)	<0.0001
Cardiac disorder	2.1 (2.1-2.2)	<0.0001	5.5 (3.5-8.5)	4.4 (2.8-6.8)	4.7 (3.8-6.0)	3.8 (3.3-4.3)	3.1 (2.9-3.3)	2.1 (2.0-2.2)	1.9 (1.8-2.0)	1.9 (1.8-1.9)	<0.0001
Diabetes	2.5 (2.4-2.6)	<0.0001	8.8 (5.7-13.3)	6.5 (4.5-9.2)	6.2 (5.1-7.5)	5.6 (4.9-6.4)	4.8 (4.4-5.3)	3.0 (2.8-3.2)	2.2 (2.1-2.3)	1.7 (1.6-1.8)	<0.0001
Hypertension	1.0 (0.9-1.2)	0.72	18.7 (6.4-54.1)	2.3 (0.3-16.7)	2.5 (1.2-5.4)	1.5 (0.9-2.5)	1.4 (1.0-1.9)	0.7 (0.5-1.0)	0.8 (0.6-1.0)	1.2 (0.9-1.7)	<0.0001
Immune deficiency disorder	3.6 (2.9-4.4)	<0.0001	5.9 (1.8-19.1)	2.5 (0.8-8.0)	6.1 (3.4-11.1)	5.1 (3.1-8.4)	4.5 (3.1-6.4)	2.7 (1.7-4.3)	3.1 (1.8-5.2)	0.9 (0.4-2.2)	0.009
Kidney disease	4.9 (4.4-5.4)	<0.0001	9.9 (4.5-21.9)	9.6 (4.8-19.0)	9.7 (6.4-14.9)	8.8 (6.3-12.2)	7.6 (6.0-9.7)	5.0 (4.0-6.2)	3.5 (2.9-4.4)	3.0 (2.4-3.7)	<0.0001
Liver disease	2.1 (1.8-2.5)	<0.0001	10.6 (2.4-46.6)	4.7 (1.4-15.4)	3.2 (1.6-6.3)	4.5 (3.1-6.5)	2.2 (1.6-3.0)	2.4 (1.8-3.2)	1.3 (0.8-2.0)	0.7 (0.4-1.2)	<0.0001
Lung disease	2.2 (2.1-2.3)	<0.0001	1.6 (1.1-2.3)	1.9 (1.4-2.7)	2.6 (2.1-3.4)	2.7 (2.2-3.2)	2.6 (2.2-3.0)	2.4 (2.1-2.7)	2.0 (1.8-2.3)	1.7 (1.5-2.0)	<0.0001
Neurological disorders	2.2 (2.0-2.4)	<0.0001	8.3 (5.0-13.9)	5.8 (3.4-9.9)	5.2 (3.5-7.6)	4.9 (3.6-6.7)	4.0 (3.1-5.1)	3.2 (2.6-4.0)	1.7 (1.4-2.1)	1.5 (1.3-1.7)	<0.0001
Obesity	1.8 (1.4-2.3)	<0.0001	1.2 (0.2-8.6)	3.1 (1.7-5.9)	1.1 (0.5-2.4)	2.0 (1.3-3.3)	2.1 (1.4-3.0)	1.3 (0.8-2.2)	1.6 (0.9-3.0)	2.5 (1.2-5.3)	0.29
Any underlying condition (≥1)	2.3 (2.3-2.4)	<0.0001	5.2 (4.4-6.0)	3.1 (2.7-3.6)	3.8 (3.4-4.2)	3.9 (3.6-4.2)	3.3 (3.2-3.5)	2.4 (2.4-2.5)	2.0 (1.9-2.1)	1.8 (1.7-1.8)	<0.0001
Number of underlying conditions (1)	2.3 (2.3-2.3)	<0.0001	5.1 (4.3-6.0)	3.1 (2.7-3.6)	3.8 (3.4-4.2)	3.8 (3.5-4.1)	3.3 (3.2-3.5)	2.4 (2.3-2.5)	2.0 (1.9-2.0)	1.7 (1.7-1.8)	<0.0001
Number of underlying conditions (2)	2.6 (2.4-2.8)	<0.0001	6.0 (3.5-10.4)	4.2 (2.8-6.4)	4.2 (3.1-5.8)	4.5 (3.7-5.5)	3.0 (2.6-3.5)	2.4 (2.1-2.7)	2.2 (1.9-2.4)	2.4 (2.1-2.7)	<0.0001
Number of underlying conditions (3+)	4.6 (4.2-5.0)	<0.0001	28.2 (10.4-76.1)	10.6 (4.1-27.3)	8.8 (4.8-16.0)	9.3 (6.6-12.9)	6.3 (5.1-7.7)	4.9 (4.1-5.7)	4.7 (4.1-5.4)	3.1 (2.7-3.6)	<0.0001

[1]p-values based on likelihood ratio test; aOR: adjusted odds ratio; CI: confidence interval; NA: Not available as no outcome occurred within the age group; Age presented in years

Table S11 Age-adjusted and age-stratum specific associations between underlying condition and death for the sensitivity analysis, 6 February – 13 December 2020

	Age-adjusted model		Age-interaction model, stratum-specific aOR (95% CI)								
	aOR	p-value[1]	<20	20-29	30-39	40-49	50-59	60-69	70-79	≥80	Interaction p-value[1]
Asthma	0.9 (0.4-2.4)	0.88	NA	NA	NA	NA	NA	1.7 (0.4-7.0)	1.6 (0.4-6.8)	0.4 (0.1-3.5)	0.88
Cancer	1.9 (1.8-2.0)	<0.0001	438.6 (145.5-1322.5)	69.3 (8.1-595.7)	33.5 (14.8-75.9)	18.9 (12.0-29.6)	8.8 (6.8-11.4)	5.1 (4.4-5.9)	2.1 (1.9-2.4)	1.2 (1.1-1.4)	<0.0001
Cardiac disorder	1.9 (1.9-2.0)	<0.0001	NA	52.2 (6.1-448.3)	24.6 (10.9-55.7)	7.7 (4.7-12.5)	4.5 (3.6-5.7)	3.1 (2.7-3.5)	2.1 (1.9-2.3)	1.7 (1.6-1.8)	<0.0001
Diabetes	2.0 (1.9-2.1)	<0.0001	NA	NA	7.8 (2.4-25.5)	9.5 (5.7-15.9)	8.3 (6.5-10.6)	4.0 (3.5-4.6)	2.5 (2.2-2.7)	1.5 (1.4-1.6)	<0.0001
Hypertension	0.4 (0.3-0.8)	0.0014	NA	NA	NA	NA	NA	0.2 (0.0-1.2)	0.6 (0.2-1.3)	0.5 (0.3-1.0)	0.89
Immune deficiency disorder	4.6 (3.1-6.9)	<0.0001	NA	NA	27.1 (3.7-200.6)	18.0 (4.4-73.8)	8.4 (3.1-22.7)	6.7 (3.2-14.0)	4.4 (2.3-8.4)	1.4 (0.5-3.7)	0.067
Kidney disease	4.1 (3.5-4.8)	<0.0001	NA	NA	57.0 (17.3-188.3)	15.2 (4.8-48.3)	17.7 (10.4-30.2)	10.3 (7.5-14.2)	4.1 (3.1-5.3)	2.5 (2.0-3.1)	<0.0001
Liver disease	2.3 (1.7-3.2)	<0.0001	NA	NA	42.8 (10.2-180.4)	7.8 (1.9-31.8)	7.6 (3.9-14.9)	4.3 (2.6-7.1)	1.1 (0.5-2.4)	1.0 (0.5-1.9)	<0.0001
Lung disease	2.1 (1.9-2.4)	<0.0001	NA	14.5 (1.7-124.4)	14.0 (5.9-33.5)	3.4 (1.4-8.3)	3.6 (2.2-5.7)	3.4 (2.6-4.5)	2.3 (1.9-2.8)	1.7 (1.4-1.9)	<0.0001
Neurological disorders	3.1 (2.8-3.5)	<0.0001	127.6 (26.3-619.1)	195.8 (37.7-1016.8)	19.4 (4.6-81.2)	25.5 (12.3-52.9)	17.0 (10.7-27.1)	8.0 (5.7-11.2)	3.6 (2.9-4.6)	2.3 (2.0-2.7)	<0.0001
Obesity	5.6 (2.3-14.0)	0.00017	NA	NA	NA	NA	10.6 (2.9-39.1)	8.5 (2.5-29.1)	4.2 (1.0-17.2)	4.0 (1.1-15.0)	0.78
Any underlying condition (≥1)	2.1 (2.1-2.2)	<0.0001	33.8 (12.6-90.7)	24.0 (7.3-78.8)	16.6 (10.5-26.4)	9.4 (7.0-12.4)	6.5 (5.6-7.7)	4.1 (3.7-4.5)	2.3 (2.2-2.5)	1.6 (1.5-1.7)	<0.0001
Number of underlying conditions (1)	2.1 (2.0-2.1)	<0.0001	33.8 (12.3-93.3)	23.5 (6.8-81.3)	16.4 (10.2-26.4)	9.4 (7.0-12.6)	6.4 (5.4-7.5)	3.9 (3.6-4.3)	2.3 (2.1-2.4)	1.6 (1.5-1.6)	<0.0001
Number of underlying conditions (2)	3.0 (2.7-3.4)	<0.0001	63.3 (7.7-518.5)	60.7 (7.1-522.3)	34.7 (13.5-89.3)	9.6 (3.9-23.7)	9.7 (6.3-15.0)	5.6 (4.4-7.3)	2.6 (2.1-3.2)	2.4 (2.1-2.8)	<0.0001
Number of underlying conditions (3+)	5.5 (4.8-6.3)	<0.0001	NA	NA	NA	32.0 (10.0-102.1)	18.7 (10.3-33.7)	12.6 (9.4-16.8)	6.5 (5.3-8.0)	3.8 (3.2-4.5)	<0.0001

[1]p-values based on likelihood ratio test; aOR: adjusted odds ratio; CI: confidence interval; NA: Not available as no outcome occurred within the age group; Age presented in years

Table S12 Age-adjusted and age-stratum specific associations between underlying condition and in-hospital death for the sensitivity analysis, 6 February – 13 December 2020

	Age-adjusted model		Age-interaction model, stratum-specific aOR (95% CI)								
	**aOR**	**p-value[1]**	**<20**	**20-29**	**30-39**	**40-49**	**50-59**	**60-69**	**70-79**	**≥80**	**Interaction p-value[1]**
Asthma	0.7 (0.2-2.0)	0.46	NA	NA	NA	NA	NA	2.3 (0.5-11.0)	0.5 (0.1-4.6)	0.5 (0.1-4.2)	0.82
Cancer	1.5 (1.4-1.7)	<0.0001	20.5 (5.7-73.8)	14.9 (1.6-140.3)	11.3 (4.4-29.3)	7.8 (4.7-12.9)	4.3 (3.2-5.7)	2.6 (2.2-3.1)	1.5 (1.3-1.7)	1.1 (0.9-1.2)	<0.0001
Cardiac disorder	1.5 (1.4-1.6)	<0.0001	NA	16.3 (1.7-152.9)	7.0 (2.7-17.7)	2.5 (1.5-4.2)	1.8 (1.4-2.3)	1.8 (1.6-2.1)	1.5 (1.4-1.7)	1.4 (1.3-1.5)	0.00047
Diabetes	1.5 (1.4-1.6)	<0.0001	NA	NA	2.0 (0.6-6.9)	2.2 (1.3-3.9)	2.5 (1.9-3.2)	1.9 (1.6-2.2)	1.6 (1.4-1.8)	1.3 (1.2-1.4)	<0.0001
Hypertension	0.5 (0.3-0.9)	0.021	NA	NA	NA	NA	NA	0.2 (0.0-1.5)	0.8 (0.3-2.1)	0.5 (0.2-1.3)	0.87
Immune deficiency disorder	3.1 (1.9-4.9)	<0.0001	NA	NA	9.1 (1.1-75.0)	2.8 (0.4-21.2)	2.9 (1.0-8.3)	4.2 (1.8-9.8)	3.0 (1.4-6.5)	1.3 (0.3-5.7)	0.9
Kidney disease	2.4 (2.0-2.9)	<0.0001	NA	NA	10.7 (3.0-38.3)	2.9 (0.9-9.6)	3.9 (2.1-7.1)	3.8 (2.6-5.5)	2.1 (1.6-2.9)	1.8 (1.4-2.4)	0.024
Liver disease	1.7 (1.1-2.6)	0.016	NA	NA	32.4 (6.0-173.9)	2.9 (0.7-12.6)	3.7 (1.5-8.9)	2.4 (1.3-4.4)	0.5 (0.1-1.5)	0.8 (0.3-2.3)	0.0056
Lung disease	1.6 (1.4-1.8)	<0.0001	NA	NA	9.1 (3.6-23.5)	1.4 (0.5-3.8)	1.7 (1.0-2.8)	2.1 (1.5-2.8)	1.5 (1.2-1.9)	1.4 (1.2-1.8)	0.048
Neurological disorders	3.1 (2.6-3.6)	<0.0001	26.5 (4.6-151.9)	23.6 (2.5-226.7)	2.9 (0.4-22.4)	10.1 (4.5-22.7)	6.6 (3.8-11.4)	4.4 (2.9-6.6)	3.0 (2.2-4.1)	2.3 (1.9-2.9)	0.00018
Obesity	3.2 (1.1-9.4)	0.031	NA	NA	NA	NA	4.2 (0.8-21.5)	7.9 (1.8-34.6)	3.3 (0.7-16.7)	1.6 (0.3-8.2)	0.7
Any underlying condition (≥1)	1.6 (1.6-1.7)	<0.0001	8.2 (2.7-25.5)	6.6 (1.6-26.6)	6.5 (3.8-11.2)	3.2 (2.4-4.5)	2.6 (2.1-3.1)	2.2 (2.0-2.5)	1.6 (1.5-1.7)	1.4 (1.3-1.4)	<0.0001
Number of underlying conditions (1)	1.6 (1.5-1.6)	<0.0001	8.1 (2.5-25.7)	5.6 (1.2-25.3)	6.2 (3.5-10.8)	3.2 (2.3-4.4)	2.5 (2.1-2.9)	2.1 (1.9-2.3)	1.6 (1.5-1.7)	1.3 (1.3-1.4)	<0.0001
Number of underlying conditions (2)	2.3 (2.0-2.7)	<0.0001	15.7 (1.7-145.6)	24.9 (2.7-233.6)	16.6 (5.4-51.1)	3.9 (1.5-10.0)	4.5 (2.7-7.6)	4.0 (3.0-5.3)	1.9 (1.4-2.4)	1.9 (1.5-2.3)	<0.0001
Number of underlying conditions (3+)	3.3 (2.8-3.9)	<0.0001	NA	NA	NA	7.8 (2.3-26.2)	5.6 (2.9-10.6)	5.9 (4.2-8.1)	3.4 (2.6-4.3)	2.4 (1.9-3.0)	<0.0001

[1]p-values based on likelihood ratio test; aOR: adjusted odds ratio; CI: confidence interval; NA: Not available as no outcome occurred within the age group; Age presented in years

Table S13 Predicted probabilities of hospitalisation for each underlying conditions and exposure compared to COVID-19 cases without an underlying condition for the sensitivity analysis, 7 February – 13 December 2020

	Variable value*	Age groups							
		**<20**	**20-29**	**30-39**	**40-49**	**50-59**	**60-69**	**70-79**	**≥80**
Asthma	0	1.90 (1.79-2.03)	2.34 (2.22-2.48)	3.47 (3.30-3.63)	4.73 (4.53-4.93)	7.77 (7.47-8.07)	16.09 (15.54-16.67)	29.77 (28.89-30.67)	35.26 (34.30-36.23)
Asthma	1	4.19 (2.18-7.89)	2.98 (1.55-5.65)	6.47 (4.09-10.07)	9.16 (6.41-12.93)	13.58 (9.56-18.93)	18.26 (11.82-27.12)	38.91 (24.91-55.02)	56.12 (35.63-74.71)
Cancer	0	1.92 (1.81-2.05)	2.37 (2.25-2.51)	3.51 (3.35-3.68)	4.80 (4.60-5.00)	7.89 (7.59-8.19)	16.29 (15.73-16.86)	30.09 (29.21-30.98)	35.77 (34.82-36.73)
Cancer	1	33.01 (26.12-40.73)	11.35 (7.99-15.88)	13.78 (11.25-16.78)	15.08 (13.38-16.94)	19.35 (18.02-20.75)	32.18 (30.62-33.78)	44.05 (42.47-45.64)	42.64 (41.11-44.18)
Cardiac disorder	0	1.83 (1.72-1.95)	2.28 (2.17-2.41)	3.39 (3.24-3.55)	4.63 (4.45-4.83)	7.61 (7.34-7.90)	15.72 (15.19-16.25)	29.07 (28.24-29.92)	34.76 (33.86-35.67)
Cardiac disorder	1	8.32 (5.84-11.73)	9.02 (6.57-12.25)	12.70 (10.75-14.93)	13.49 (12.38-14.67)	19.03 (18.12-19.97)	28.90 (27.82-30.01)	43.78 (42.54-45.04)	45.33 (44.22-46.45)
Diabetes	0	1.83 (1.71-1.94)	2.26 (2.14-2.39)	3.36 (3.20-3.52)	4.58 (4.39-4.77)	7.52 (7.24-7.82)	15.58 (15.05-16.13)	28.91 (28.05-29.78)	34.44 (33.51-35.39)
Diabetes	1	12.62 (8.97-17.47)	10.38 (7.74-13.78)	15.69 (13.54-18.10)	18.91 (17.24-20.71)	26.00 (24.55-27.50)	35.56 (34.12-37.02)	46.20 (44.73-47.67)	44.80 (43.43-46.17)
Hypertension	0	1.92 (1.80-2.04)	2.36 (2.24-2.50)	3.50 (3.34-3.66)	4.77 (4.57-4.97)	7.83 (7.54-8.14)	16.21 (15.66-16.78)	29.96 (29.08-30.85)	35.47 (34.52-36.43)
Hypertension	1	22.09 (8.91-45.10)	5.38 (1.34-19.31)	5.84 (2.93-11.30)	5.67 (3.82-8.34)	9.01 (7.13-11.33)	12.84 (10.25-15.97)	25.07 (20.07-30.83)	37.14 (30.63-44.14)
Immune deficiency disorder	0	1.91 (1.79-2.04)	2.35 (2.23-2.49)	3.48 (3.32-3.65)	4.75 (4.55-4.95)	7.80 (7.50-8.10)	16.14 (15.58-16.72)	29.84 (28.95-30.75)	35.34 (34.38-36.32)
Immune deficiency disorder	1	13.85 (6.25-27.93)	8.30 (4.32-15.39)	12.91 (8.52-19.10)	19.03 (14.36-24.77)	22.04 (17.77-27.01)	35.35 (27.23-44.41)	50.44 (39.39-61.46)	41.47 (28.67-55.54)
Kidney disease	0	1.92 (1.80-2.04)	2.36 (2.23-2.49)	3.49 (3.33-3.66)	4.76 (4.56-4.97)	7.82 (7.52-8.12)	16.18 (15.62-16.76)	29.90 (29.01-30.81)	35.41 (34.45-36.39)
Kidney disease	1	13.22 (6.88-23.89)	18.06 (11.34-27.54)	24.19 (18.47-31.01)	27.99 (22.99-33.62)	36.63 (31.88-41.65)	45.72 (41.03-50.49)	57.96 (53.37-62.42)	58.12 (53.83-62.30)
Liver disease	0	1.92 (1.81-2.05)	2.37 (2.24-2.50)	3.50 (3.34-3.67)	4.78 (4.58-4.99)	7.85 (7.55-8.16)	16.25 (15.68-16.83)	30.02 (29.12-30.93)	35.54 (34.58-36.52)
Liver disease	1	12.14 (3.08-37.54)	5.62 (1.78-16.34)	12.68 (7.84-19.87)	16.11 (12.22-20.95)	14.07 (10.90-17.97)	30.81 (25.54-36.62)	34.59 (26.47-43.71)	32.27 (23.23-42.87)
Lung disease	0	1.93 (1.81-2.05)	2.37 (2.25-2.51)	3.51 (3.35-3.68)	4.79 (4.59-5.00)	7.87 (7.57-8.17)	16.27 (15.71-16.84)	30.03 (29.15-30.93)	35.62 (34.66-36.58)
Lung disease	1	2.65 (1.88-3.74)	4.16 (3.24-5.32)	8.09 (6.75-9.66)	9.97 (8.72-11.37)	16.79 (15.28-18.43)	31.03 (28.70-33.47)	44.81 (42.03-47.63)	45.39 (42.74-48.07)

Neurological disorders	0	1.91 (1.80-2.04)	2.36 (2.23-2.49)	3.49 (3.33-3.66)	4.76 (4.56-4.96)	7.81 (7.52-8.12)	16.16 (15.60-16.74)	29.86 (28.97-30.77)	35.39 (34.42-36.36)
Neurological disorders	1	12.24 (7.83-18.64)	9.79 (6.29-14.94)	12.96 (9.61-17.26)	16.68 (13.29-20.72)	24.53 (20.94-28.51)	33.94 (29.71-38.45)	36.92 (33.31-40.69)	38.60 (35.91-41.36)
Obesity	0	1.91 (1.79-2.03)	2.35 (2.22-2.48)	3.47 (3.31-3.64)	4.74 (4.54-4.94)	7.78 (7.49-8.09)	16.12 (15.56-16.70)	29.82 (28.93-30.72)	35.31 (34.35-36.29)
Obesity	1	5.14 (1.30-18.29)	9.06 (5.42-14.75)	6.70 (3.99-11.05)	12.29 (8.74-17.01)	18.27 (13.85-23.70)	26.14 (18.44-35.65)	48.81 (34.69-63.13)	71.19 (54.53-83.59)
Any underlying condition (≥1)	0	1.74 (1.64-1.84)	2.18 (2.08-2.29)	3.24 (3.11-3.37)	4.42 (4.27-4.57)	7.29 (7.08-7.50)	15.06 (14.67-15.47)	28.04 (27.40-28.69)	33.81 (33.12-34.50)
Any underlying condition (≥1)	1	7.72 (6.84-8.69)	6.42 (5.80-7.11)	10.76 (10.09-11.48)	13.83 (13.25-14.44)	19.47 (18.92-20.04)	30.50 (29.83-31.19)	44.02 (43.26-44.78)	44.43 (43.72-45.15)
Number of underlying conditions	0	1.65 (1.56-1.75)	2.07 (1.97-2.18)	3.08 (2.96-3.21)	4.20 (4.05-4.35)	6.91 (6.71-7.13)	14.34 (13.95-14.75)	26.84 (26.19-27.50)	32.40 (31.70-33.11)
Number of underlying conditions	1	7.15 (6.29-8.12)	5.99 (5.36-6.69)	10.06 (9.38-10.79)	12.82 (12.23-13.44)	18.41 (17.84-19.00)	28.93 (28.21-29.65)	41.80 (40.97-42.62)	42.35 (41.58-43.13)
Number of underlying conditions	2	10.02 (6.67-14.79)	8.87 (6.61-11.82)	11.93 (9.61-14.72)	15.90 (13.86-18.17)	18.41 (16.71-20.24)	28.34 (26.38-30.38)	44.09 (41.75-46.46)	47.76 (45.48-50.04)
Number of underlying conditions	3+	30.40 (15.21-51.54)	15.67 (7.65-29.41)	31.39 (23.41-40.64)	29.84 (24.77-35.46)	32.40 (28.89-36.11)	46.78 (43.50-50.09)	61.75 (58.92-64.51)	54.52 (51.89-57.12)

*0 indicates no underlying health condition, reference group; The probabilities were estimated from the fully adjusted age-interaction models. Baseline risks may therefore vary between the different models. CI: Confidence interval; NA: Not available as no outcome occurred within the age group, PP: predicted probabilities.

Table S14 Predicted probabilities of death for each underlying conditions and exposure compared to COVID-19 cases without an underlying condition for the sensitivity analysis, 7 February – 13 December 2020

	Variable value*	Age groups							
Variable		**<20**	**20-29**	**30-39**	**40-49**	**50-59**	**60-69**	**70-79**	**≥80**
Asthma	0	0.00 (0.00-0.01)	0.01 (0.00-0.01)	0.03 (0.02-0.04)	0.07 (0.06-0.09)	0.22 (0.18-0.26)	0.98 (0.84-1.15)	3.75 (3.24-4.34)	10.83 (9.47-12.36)
Asthma	1	NA	NA	NA	NA	NA	1.19 (0.29-4.73)	4.06 (0.96-15.52)	8.29 (1.95-29.11)
Cancer	0	0.01 (0.00-0.01)	0.01 (0.01-0.02)	0.04 (0.03-0.05)	0.08 (0.07-0.10)	0.26 (0.22-0.30)	1.17 (1.04-1.33)	4.48 (3.99-5.02)	12.78 (11.53-14.14)
Cancer	1	2.02 (0.89-4.49)	0.44 (0.11-1.77)	1.31 (0.77-2.24)	1.60 (1.18-2.18)	2.44 (2.02-2.93)	5.41 (4.69-6.22)	9.69 (8.59-10.92)	16.24 (14.57-18.05)
Cardiac disorder	0	0.01 (0.00-0.01)	0.01 (0.00-0.02)	0.04 (0.03-0.05)	0.08 (0.07-0.10)	0.25 (0.22-0.28)	1.12 (1.00-1.25)	4.28 (3.87-4.73)	12.31 (11.26-13.44)
Cardiac disorder	1	0.32 (0.08-1.29)	0.15 (0.02-1.04)	0.59 (0.32-1.11)	0.59 (0.43-0.81)	1.02 (0.86-1.20)	3.07 (2.72-3.46)	8.78 (7.94-9.69)	18.76 (17.25-20.37)
Diabetes	0	0.00 (0.00-0.01)	0.01 (0.00-0.01)	0.03 (0.02-0.04)	0.07 (0.05-0.08)	0.20 (0.17-0.24)	0.92 (0.77-1.09)	3.50 (2.97-4.12)	10.12 (8.69-11.75)
Diabetes	1	NA	NA	0.24 (0.10-0.57)	0.66 (0.45-0.97)	1.56 (1.25-1.95)	3.60 (3.01-4.31)	8.10 (6.88-9.52)	14.48 (12.47-16.75)
Hypertension	0	0.00 (0.00-0.01)	0.01 (0.00-0.01)	0.03 (0.02-0.04)	0.07 (0.05-0.08)	0.20 (0.17-0.24)	0.92 (0.78-1.09)	3.52 (3.00-4.13)	10.21 (8.81-11.81)
Hypertension	1	NA	NA	NA	NA	NA	0.35 (0.11-1.10)	1.89 (0.88-4.02)	14.55 (10.03-20.66)
Immune deficiency disorder	0	0.00 (0.00-0.01)	0.01 (0.00-0.01)	0.03 (0.02-0.04)	0.07 (0.06-0.09)	0.21 (0.18-0.25)	0.96 (0.82-1.13)	3.68 (3.15-4.29)	10.63 (9.22-12.23)
Immune deficiency disorder	1	NA	NA	1.06 (0.33-3.30)	0.69 (0.22-2.15)	1.54 (0.83-2.84)	4.81 (2.69-8.46)	10.49 (6.34-16.88)	22.43 (13.71-34.47)
Kidney disease	0	0.01 (0.00-0.01)	0.01 (0.00-0.01)	0.03 (0.02-0.05)	0.07 (0.06-0.09)	0.22 (0.19-0.26)	1.02 (0.88-1.18)	3.88 (3.38-4.45)	11.17 (9.85-12.65)
Kidney disease	1	NA	NA	1.05 (0.39-2.82)	1.30 (0.64-2.64)	2.71 (1.77-4.15)	7.25 (5.49-9.50)	13.92 (11.25-17.11)	21.84 (18.36-25.78)
Liver disease	0	0.00 (0.00-0.01)	0.01 (0.00-0.01)	0.03 (0.02-0.04)	0.07 (0.06-0.09)	0.21 (0.18-0.25)	0.96 (0.81-1.13)	3.66 (3.12-4.28)	10.59 (9.15-12.22)
Liver disease	1	NA	NA	0.79 (0.19-3.15)	0.49 (0.16-1.54)	1.55 (0.89-2.67)	3.59 (2.29-5.61)	4.52 (2.48-8.12)	11.91 (7.47-18.46)
Lung disease	0	0.01 (0.00-0.01)	0.01 (0.00-0.01)	0.03 (0.03-0.05)	0.07 (0.06-0.09)	0.23 (0.19-0.27)	1.04 (0.90-1.20)	3.97 (3.46-4.56)	11.46 (10.11-12.96)
Lung disease	1	NA	0.04 (0.01-0.28)	0.32 (0.16-0.65)	0.34 (0.19-0.59)	0.71 (0.49-1.02)	3.13 (2.48-3.95)	9.24 (7.74-10.99)	18.66 (16.18-21.43)

Neurological disorders	0	0.00 (0.00-0.01)	0.01 (0.00-0.01)	0.03 (0.02-0.04)	0.07 (0.06-0.09)	0.22 (0.18-0.27)	1.01 (0.84-1.20)	3.86 (3.26-4.56)	11.10 (9.52-12.91)
Neurological disorders	1	0.64 (0.16-2.55)	0.54 (0.13-2.17)	0.66 (0.25-1.79)	1.46 (0.81-2.63)	3.87 (2.72-5.48)	6.53 (4.84-8.75)	12.72 (10.26-15.68)	23.77 (20.33-27.61)
Obesity	0	0.00 (0.00-0.01)	0.01 (0.00-0.01)	0.03 (0.02-0.04)	0.07 (0.06-0.09)	0.21 (0.18-0.25)	0.97 (0.83-1.13)	3.70 (3.21-4.27)	10.70 (9.39-12.18)
Obesity	1	NA	NA	0.44 (0.06-3.12)	0.78 (0.19-3.13)	1.83 (0.67-4.94)	7.46 (2.95-17.62)	13.59 (4.21-36.02)	36.67 (14.71-66.04)
Any underlying condition (≥1)	0	0.01 (0.00-0.01)	0.01 (0.01-0.02)	0.04 (0.03-0.06)	0.10 (0.08-0.12)	0.30 (0.27-0.34)	1.39 (1.29-1.50)	5.34 (5.03-5.66)	15.03 (14.35-15.74)
Any underlying condition (≥1)	1	0.23 (0.13-0.42)	0.10 (0.05-0.21)	0.60 (0.47-0.77)	0.87 (0.74-1.01)	1.83 (1.69-1.99)	4.98 (4.70-5.29)	11.72 (11.16-12.29)	22.30 (21.43-23.21)
Number of underlying conditions	0	0.01 (0.00-0.01)	0.01 (0.01-0.02)	0.04 (0.03-0.05)	0.08 (0.07-0.10)	0.26 (0.23-0.29)	1.19 (1.10-1.29)	4.55 (4.27-4.85)	12.93 (12.26-13.62)
Number of underlying conditions	1	0.22 (0.12-0.40)	0.08 (0.04-0.19)	0.50 (0.38-0.66)	0.77 (0.66-0.91)	1.56 (1.42-1.70)	4.16 (3.89-4.44)	9.87 (9.33-10.44)	18.87 (17.98-19.79)
Number of underlying conditions	2	0.24 (0.03-1.68)	0.27 (0.07-1.06)	0.93 (0.48-1.78)	0.61 (0.32-1.18)	1.46 (1.04-2.03)	5.47 (4.59-6.50)	11.50 (10.10-13.07)	29.35 (27.17-31.63)
Number of underlying conditions	≥3	NA	NA	2.11 (0.68-6.39)	1.79 (0.80-3.94)	4.66 (3.34-6.46)	9.37 (7.70-11.36)	19.58 (17.35-22.02)	34.92 (32.27-37.66)

*0 indicates no underlying health condition, reference group; The probabilities were estimated from the fully adjusted age-interaction models. Baseline risks may therefore vary between the different models. CI: Confidence interval; NA: Not available as no outcome occurred within the age group, PP: predicted probabilities.

Table S15 Predicted probabilities of in-hospital death for each underlying conditions and exposure compared to COVID-19 cases without an underlying condition for the sensitivity analysis, 7 February – 13 December 2020

	Variable value*	Age groups							
		<20	20-29	30-39	40-49	50-59	60-69	70-79	≥80
Asthma	0	0.21 (0.09-0.51)	0.29 (0.15-0.56)	0.61 (0.42-0.87)	1.09 (0.85-1.40)	2.02 (1.66-2.46)	4.94 (4.15-5.88)	10.41 (8.86-12.19)	22.09 (19.22-25.26)
Asthma	1	NA	NA	NA	NA	NA	10.63 (2.52-35.32)	4.50 (0.56-28.18)	22.82 (5.70-59.14)
Cancer	0	0.25 (0.10-0.61)	0.35 (0.19-0.66)	0.73 (0.51-1.04)	1.32 (1.06-1.65)	2.46 (2.08-2.89)	6.00 (5.22-6.89)	12.52 (11.05-14.15)	25.88 (23.33-28.60)
Cancer	1	5.61 (2.28-13.16)	4.13 (1.00-15.50)	8.86 (5.02-15.16)	9.42 (6.81-12.88)	10.90 (8.94-13.22)	14.28 (12.27-16.55)	19.54 (17.22-22.10)	29.85 (26.77-33.13)
Cardiac disorder	0	0.24 (0.10-0.58)	0.34 (0.18-0.63)	0.70 (0.50-0.99)	1.26 (1.02-1.56)	2.35 (2.02-2.74)	5.74 (5.07-6.50)	12.00 (10.75-13.38)	25.04 (22.85-27.36)
Cardiac disorder	1	1.81 (0.25-11.98)	1.41 (0.19-9.50)	3.32 (1.69-6.40)	3.16 (2.24-4.45)	4.08 (3.40-4.89)	8.67 (7.59-9.88)	17.87 (16.06-19.83)	33.16 (30.52-35.90)
Diabetes	0	0.20 (0.08-0.50)	0.28 (0.15-0.54)	0.59 (0.40-0.85)	1.06 (0.82-1.36)	1.96 (1.60-2.41)	4.82 (4.02-5.78)	10.17 (8.59-12.00)	21.62 (18.68-24.89)
Diabetes	1	NA	NA	1.38 (0.56-3.35)	2.86 (1.92-4.24)	5.03 (3.97-6.34)	9.10 (7.55-10.95)	15.93 (13.51-18.70)	26.69 (23.13-30.57)
Hypertension	0	0.20 (0.08-0.49)	0.28 (0.15-0.54)	0.58 (0.40-0.84)	1.05 (0.82-1.35)	1.95 (1.59-2.39)	4.78 (3.98-5.72)	10.07 (8.51-11.88)	21.46 (18.55-24.70)
Hypertension	1	NA	NA	NA	NA	NA	1.98 (0.47-7.90)	7.41 (2.92-17.56)	18.76 (10.13-32.14)
Immune deficiency disorder	0	0.20 (0.08-0.50)	0.29 (0.15-0.55)	0.59 (0.41-0.86)	1.07 (0.83-1.38)	1.98 (1.61-2.43)	4.84 (4.01-5.83)	10.20 (8.58-12.09)	21.71 (18.68-25.08)
Immune deficiency disorder	1	NA	NA	4.77 (1.12-18.14)	1.90 (0.46-7.55)	4.41 (2.19-8.68)	12.98 (7.05-22.67)	18.66 (10.75-30.41)	40.99 (23.00-61.75)
Kidney disease	0	0.22 (0.09-0.54)	0.31 (0.16-0.59)	0.65 (0.45-0.93)	1.16 (0.92-1.48)	2.16 (1.79-2.59)	5.27 (4.48-6.18)	11.03 (9.52-12.75)	23.22 (20.47-26.20)
Kidney disease	1	NA	NA	4.08 (1.48-10.74)	3.34 (1.46-7.48)	6.83 (4.33-10.60)	15.06 (11.24-19.88)	23.02 (18.45-28.32)	34.84 (29.24-40.89)
Liver disease	0	0.21 (0.09-0.51)	0.29 (0.15-0.56)	0.61 (0.42-0.88)	1.10 (0.85-1.41)	2.03 (1.66-2.48)	4.97 (4.16-5.94)	10.47 (8.88-12.31)	22.20 (19.25-25.46)
Liver disease	1	NA	NA	7.31 (1.68-26.69)	3.19 (0.99-9.76)	8.66 (4.44-16.20)	11.09 (6.69-17.84)	9.44 (4.47-18.83)	22.47 (12.10-37.88)
Lung disease	0	0.21 (0.09-0.51)	0.29 (0.15-0.56)	0.61 (0.42-0.88)	1.10 (0.85-1.41)	2.04 (1.67-2.49)	4.99 (4.18-5.95)	10.51 (8.92-12.33)	22.33 (19.40-25.57)
Lung disease	1	NA	NA	3.62 (1.75-7.32)	2.49 (1.37-4.49)	3.00 (2.01-4.45)	8.49 (6.55-10.95)	17.26 (14.13-20.91)	31.84 (27.23-36.83)

Neurological disorders	0	0.21 (0.09-0.51)	0.29 (0.15-0.56)	0.61 (0.42-0.88)	1.09 (0.85-1.41)	2.03 (1.65-2.50)	4.97 (4.12-5.98)	10.44 (8.78-12.36)	22.10 (19.03-25.51)
Neurological disorders	1	6.55 (1.52-24.17)	3.19 (0.43-20.05)	3.35 (1.03-10.29)	8.49 (4.48-15.48)	12.58 (8.44-18.33)	16.80 (12.09-22.85)	25.43 (20.09-31.63)	39.56 (33.78-45.65)
Obesity	0	0.20 (0.08-0.50)	0.29 (0.15-0.54)	0.59 (0.41-0.85)	1.07 (0.83-1.37)	1.98 (1.63-2.41)	4.84 (4.07-5.77)	10.21 (8.69-11.96)	21.73 (18.90-24.86)
Obesity	1	NA	NA	9.44 (1.29-45.43)	10.66 (2.69-33.99)	10.04 (3.16-27.62)	32.21 (13.33-59.48)	29.28 (9.11-63.13)	34.10 (10.72-69.04)
Any underlying condition (≥1)	0	0.29 (0.12-0.68)	0.39 (0.21-0.73)	0.81 (0.58-1.13)	1.49 (1.23-1.80)	2.80 (2.49-3.15)	6.82 (6.27-7.41)	14.12 (13.22-15.08)	28.66 (27.27-30.09)
Any underlying condition (≥1)	1	2.61 (1.35-4.97)	1.27 (0.57-2.82)	4.74 (3.67-6.12)	4.86 (4.14-5.70)	7.00 (6.39-7.67)	12.95 (12.13-13.81)	21.82 (20.71-22.98)	36.54 (35.09-38.02)
Number of underlying conditions	0	0.25 (0.11-0.61)	0.34 (0.18-0.64)	0.71 (0.51-0.99)	1.29 (1.06-1.57)	2.41 (2.14-2.73)	5.91 (5.41-6.46)	12.35 (11.48-13.27)	25.55 (24.13-27.03)
Number of underlying conditions	1	2.35 (1.17-4.67)	0.88 (0.33-2.33)	3.82 (2.86-5.07)	4.26 (3.59-5.06)	5.89 (5.32-6.52)	10.80 (10.01-11.64)	18.91 (17.76-20.11)	32.51 (30.92-34.14)
Number of underlying conditions	2	3.22 (0.44-19.93)	4.12 (1.02-15.17)	9.48 (4.74-18.05)	4.51 (2.35-8.51)	7.47 (5.20-10.61)	19.08 (16.01-22.58)	23.19 (20.19-26.49)	40.71 (37.28-44.24)
Number of underlying conditions	≥3	NA	NA	8.54 (2.75-23.55)	5.84 (2.43-13.38)	14.07 (9.98-19.47)	21.71 (17.91-26.07)	30.47 (26.92-34.27)	45.34 (41.48-49.26)

*0 indicates no underlying health condition, reference group; The probabilities were estimated from the fully adjusted age-interaction models. Baseline risks may therefore vary between the different models. CI: Confidence interval; NA: Not available as no outcome occurred within the age group, PP: predicted probabilities.

Figure S1 Predicted probabilities of hospitalisation, death and in-hospital death for each underlying conditions compared to COVID-19 cases without an underlying condition for the sensitivity analysis, 7 February – 13 December 2020

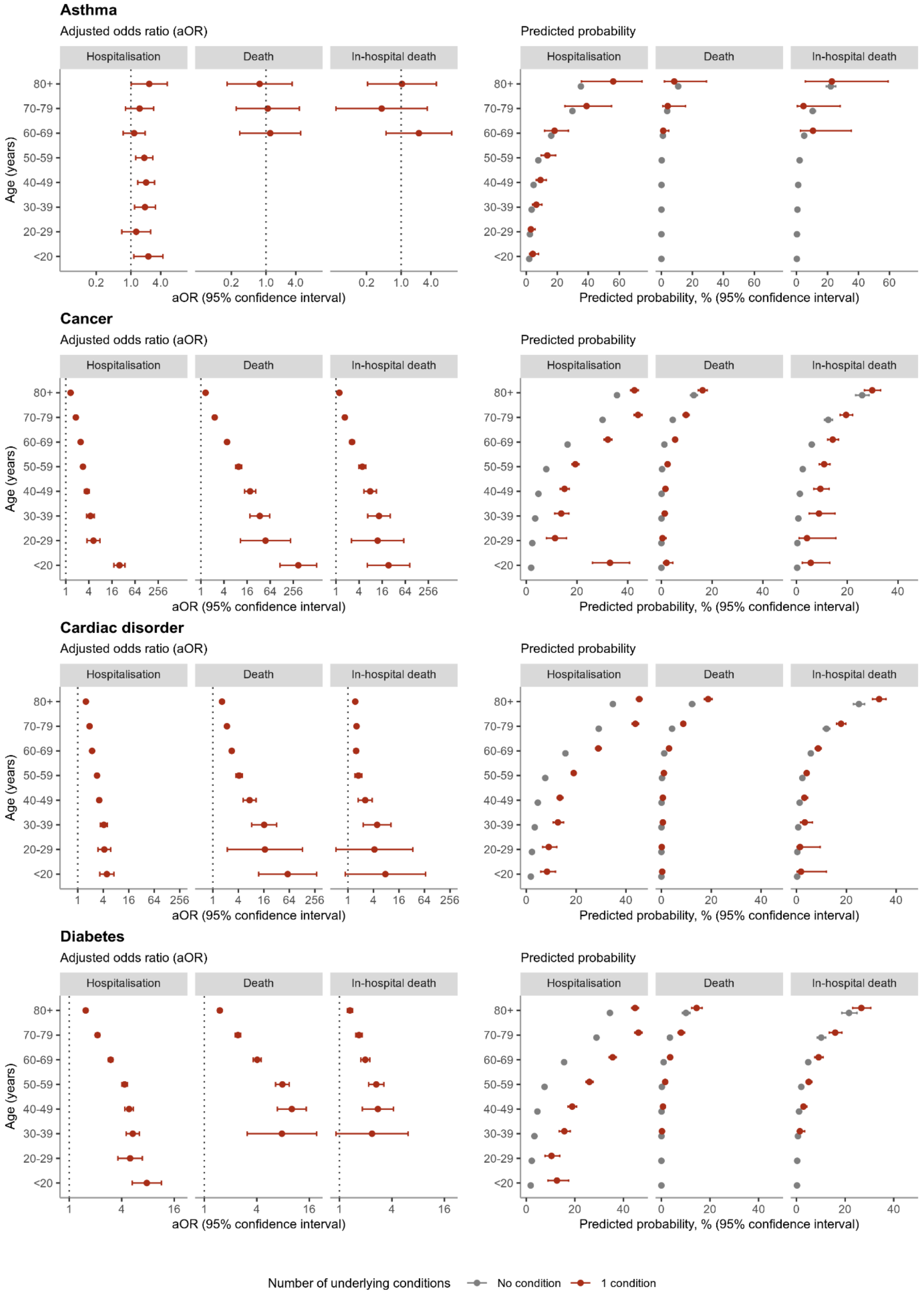

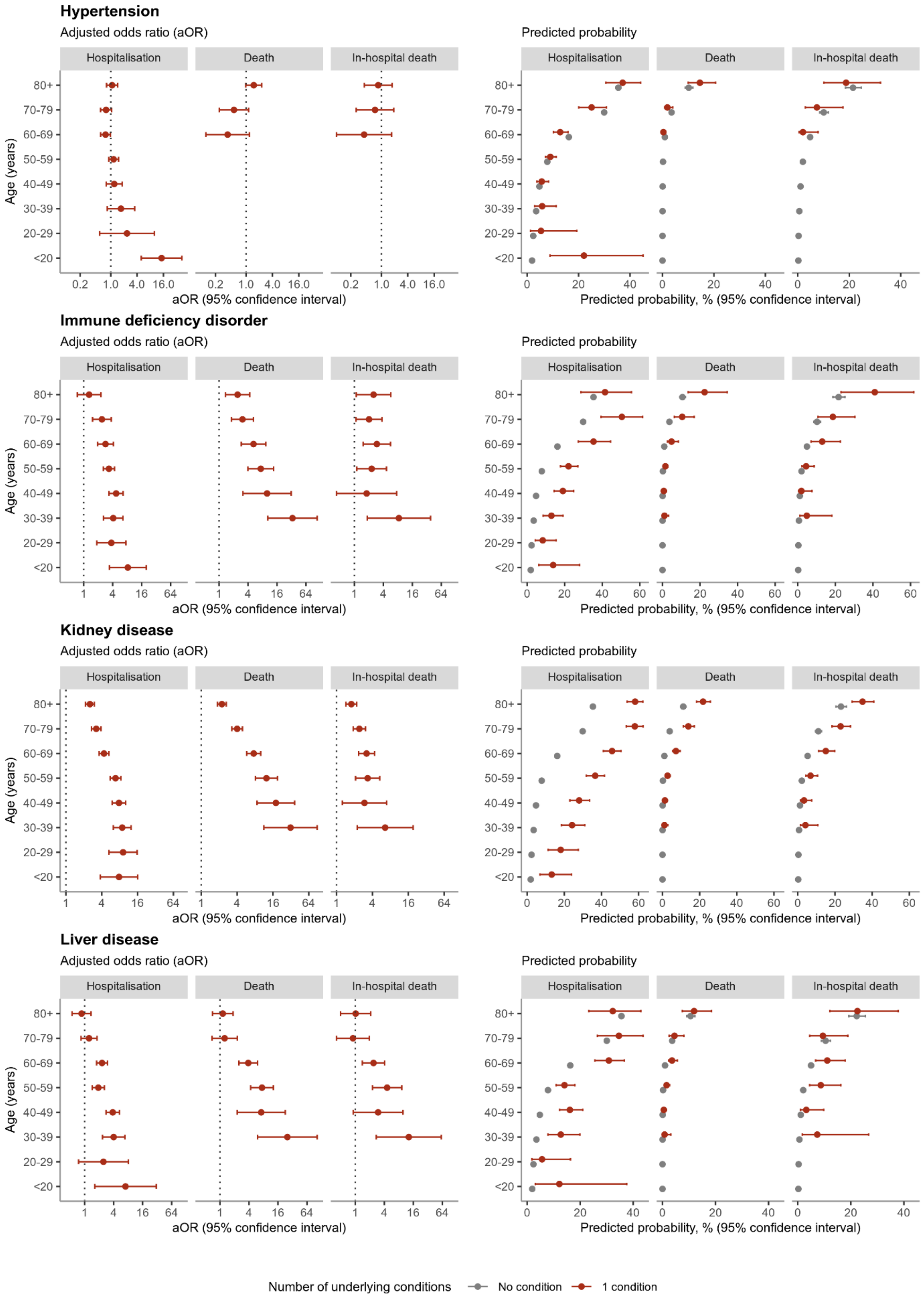
Hypertension
Adjusted odds ratio (aOR)
Predicted probability
Immune deficiency disorder
Kidney disease
Liver disease
Hospitalisation
Death
In-hospital death
Age (years)
80+
70-79
60-69
50-59
40-49
30-39
20-29
<20
0.2
1.0
4.0
16.0
1
4
16
64
0
20
40
60
aOR (95% confidence interval)
Predicted probability, % (95% confidence interval)
Number of underlying conditions
No condition
1 condition

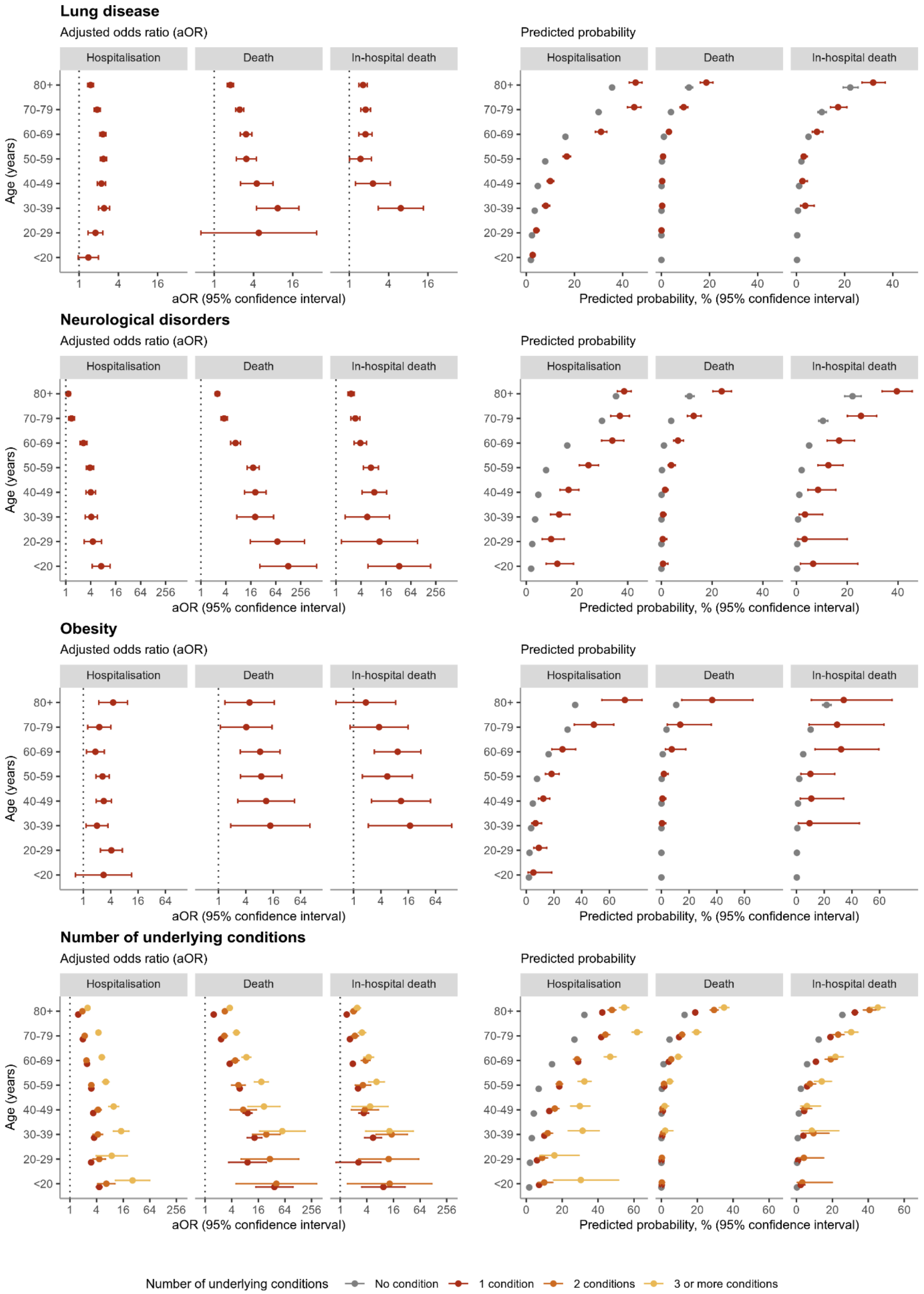

Lung disease
Adjusted odds ratio (aOR)
Predicted probability
Hospitalisation
Death
In-hospital death
Age (years)
80+
70-79
60-69
50-59
40-49
30-39
20-29
<20
aOR (95% confidence interval)
Predicted probability, % (95% confidence interval)
Neurological disorders
Obesity
Number of underlying conditions
Number of underlying conditions
No condition
1 condition
2 conditions
3 or more conditions
